# Optimizing Piezoelectric Responses: The Role of Microstructural Control and Interfacial Design in Composites

**DOI:** 10.3390/polym18212726

**Published:** 2026-11-02

**Authors:** Qinyi Liu, Xuan Liu, Xiangxiang Xu, Xue Yang

**Affiliations:** Naval University of Engineering, Wuhan 430033, China

**Keywords:** piezoelectric composites, microstructural control, piezoelectric properties, interfacial structure

## Abstract

Piezoelectric materials combine high electromechanical coupling with flexibility, making them essential in energy harvesting and smart sensing. They show great promise in human motion monitoring, self-powered sensors, vibration and noise reduction, underwater transducers, and portable power sources. However, both ceramics and polymers have inherent drawbacks when used alone. For example, ceramics are highly brittle, while polymers typically exhibit weak piezoelectric activity. Combining the two into composites can leverage their complementary strengths. Yet such composites face challenges, mainly the reduced force–electric coupling efficiency caused by poor dispersion of ceramic fillers and insufficient interfacial compatibility. This paper focuses on the fundamental piezoelectric mechanisms of piezoelectric composites and highlights microstructural control strategies as its main contribution. It provides a comprehensive review of oriented preparation methods for matrices and functional phases and examines how orientation affects piezoelectric properties. It also analyzes interface structure mechanisms through physical and chemical modification approaches. The paper further reviews application scenarios of piezoelectric materials. Overall, it offers theoretical support and practical guidance for the structural design, preparation, and engineering applications of high-performance piezoelectric composites.

## 1. Introduction

**Figure 1:**
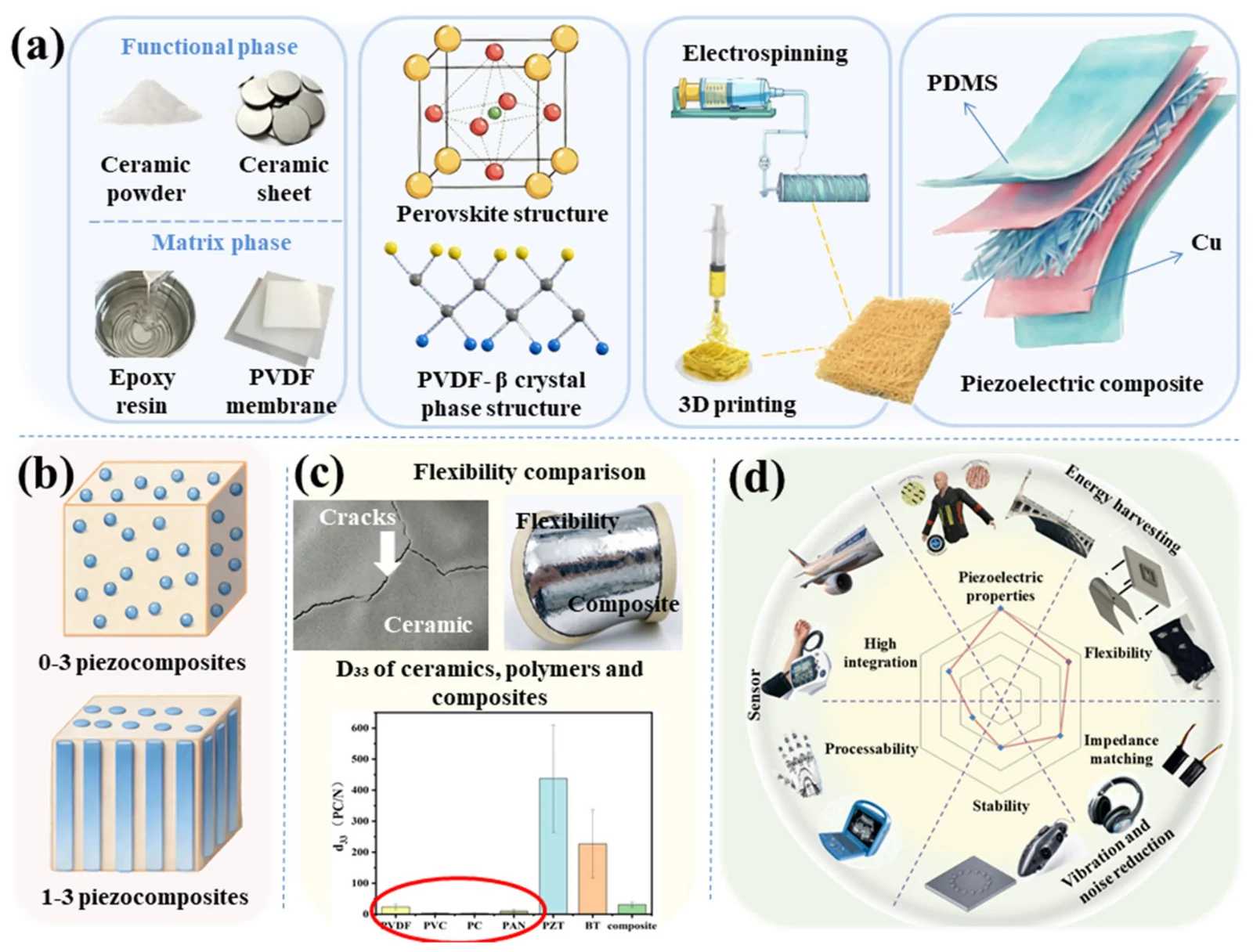
Structure, properties, and applications of piezoelectric composites: (**a**) schematic diagrams of structure and technique for piezoelectric composites [8], © 2025 Wiley-VCH GmbH; (**b**) typical Structures of piezoelectric composites; (**c**) comparison of flexibility and d_33_ values [1,9,10,11,12,13,15,16,17]; (**d**) characteristics and applications of piezoelectric composites [12,18,19,20,21,22,23,24]; Energy storing fabric, © 2015 WILEY-VCH Verlag GmbH & Co. KGaA, Weinheim. 2 Degree-of-Freedom piezoelectric Vibration Energy Harvester, © 2022 Author(s). Published under an exclusive license by AIP Publishing. SF-based sensors, © 2023 by the authors. Licensee MDPI, Basel, Switzerland. Hydrophone, © 2025 The Authors. MING LIU. 3D wood plate, © 2024 by the authors. Licensee: MDPI, Basel, Switzerland.

Piezoelectric composites are functional materials formed by combining a piezoelectric ceramic phase (e.g., lead zirconate titanate) with a polymer matrix (e.g., epoxy resin) in a specific structure. They exhibit excellent piezoelectric activity, flexibility, and structural design versatility, as shown in Figure 1a. By adjusting the bonding method, ratio, and spatial distribution of the two phases, their piezoelectric, mechanical, and acoustic properties can be tuned. Among them, the most common types are 1–3 piezocomposites, in which ceramic pillars are vertically arranged in a polymer matrix, and 0–3 piezocomposites, in which ceramic particles are randomly dispersed within a polymer matrix, as illustrated in Figure 1b [1,2,3]. Through ingenious composite design, piezoelectric composites overcome the inherent drawbacks of traditional piezoelectric ceramics, such as high brittleness and difficulty in processing and shaping, as well as the weak piezoelectric performance of pure polymers. They achieve an optimal balance between functional properties and processability and are widely used in ultrasonic transducers, sensors, and energy harvesters, as shown in Figure 1c,d [4,5,6].

However, traditional piezoelectric composites suffer from low piezoelectric coefficients, poor stress transfer efficiency, and dynamic response lag. As a result, the measured piezoelectric output is generally lower than the theoretical values, which severely limits the translation of theoretical research into practical applications. The piezoelectric properties of these composites are highly dependent on their microstructure. The random dispersion of the functional phase within the matrix prevents the effective summation of piezoelectric responses. In addition, poor interfacial compatibility introduces microscopic defects. These defects not only cause stress concentration but also lead to polarization charge loss and increased dielectric loss. As a result, stress transfer efficiency, electromechanical coupling efficiency, and overall piezoelectric performance are severely limited. To address these issues, the key technical challenge in enhancing the piezoelectric response lies in systematically controlling the microstructure and optimizing interfacial bonding to suppress defect nucleation [7]. This approach ensures the effective collection and directed transport of polarization charges while achieving synergistic enhancement of the piezoelectric response [7,8,9,10,11,12,13]. Chen et al. [14] reviewed micromechanical homogenization formalisms, including surface and interface effect modifications for heterogeneous nanostructures, accompanied by the corresponding interfacial theories for piezoelectric-polymer systems. This review gives a rigorous, single-source treatment of these topics and lays a theoretical foundation for improving the performance of piezoelectric composites. However, many experiments have explored how microstructural and interface design can boost the performance of these composites, but a systematic synthesis of the underlying mechanisms and universal strategies is still lacking.

This paper first systematically summarizes the mechanisms by which microstructure and interface engineering enhance the performance of piezoelectric composites. It then reviews fabrication approaches for the directional alignment of fillers at the microstructural level and uses representative experimental cases to illustrate how ordered structures improve piezoelectric performance. At the interface level, it generalizes two major strategies: physical modification and chemical modification. It further analyzes how specific reaction types affect piezoelectric properties, aiming to provide systematic references for material system screening and preparation method selection. Finally, it presents practical applications of such materials in sensing, energy harvesting, and transducers, and discusses prospects for multiscale design and functional integration.

## 2. Piezoelectric Behavior

### 2.1. Piezoelectric Mechanism

**Figure 2:**
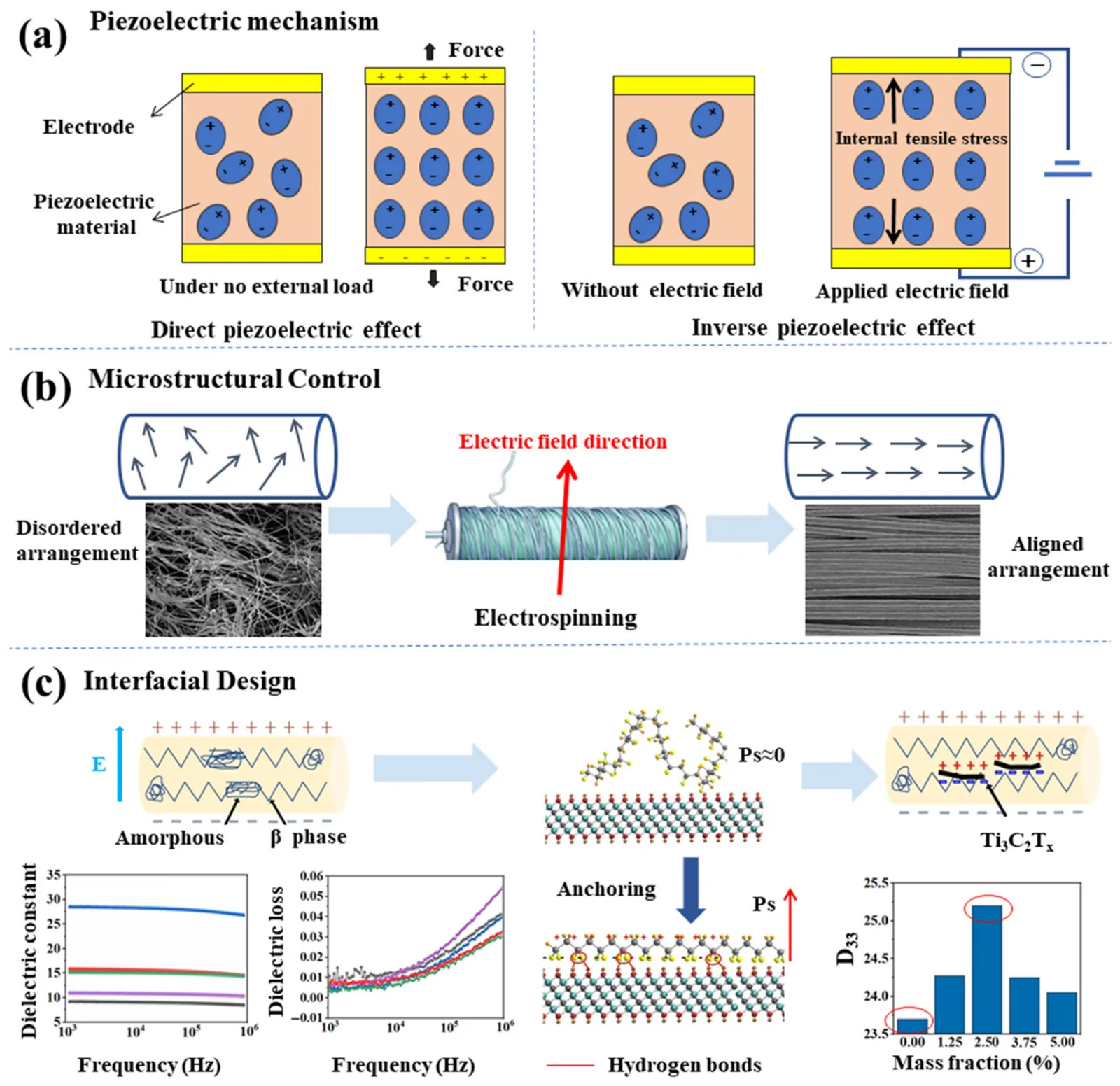
Mechanism, orientation, and interface effects on piezoelectric properties: (**a**) schematic diagram of piezoelectric mechanisms; (**b**) dipole orientation and fiber alignment; (**c**) effect of the interface structure on piezoelectric properties [29], Copyright © 2022, The Author(s).

Under normal conditions, when no external force is applied, the positive and negative charge centers in a piezoelectric material overlap, and the material appears to be electrically neutral overall. When a mechanical force is applied, opposite charges are generated within the material and accumulate at its two ends, forming an electric dipole and producing a potential difference that appears as a voltage output. This phenomenon is known as the direct piezoelectric effect, as shown in Figure 2a. Conversely, when an external electric field is applied, the positive and negative charge centers in the unit cell shift are relative to each other. This causes the crystal to deform. This phenomenon is called the inverse piezoelectric effect [13]. The constitutive Equations for the piezoelectric effect can be expressed as Equations (1) and (2) [25,26,27,28]:

*S* = *s^E^T* + *d^t^E* (1)

*D* = *d*
*T* + *ε*
*^T^E* (2)

where *S* is the strain of the piezoelectric material; *s^E^* is the elastic compliance coefficient of the piezoelectric material; *T* is the stress of the piezoelectric material; *d* is the piezoelectric strain constant; *d^t^* is the transpose of *d*; *E* is the applied electric field strength; *D* is the electric displacement; and *ε^T^* is the dielectric permittivity. All parameters refer to the piezoelectric material unless otherwise noted.

Assuming no external electric field is applied, no charge is generated by the electric field alone. The electric displacement produced by the direct piezoelectric effect is caused solely by an external force, as shown in Equation (3).

*D* = *d*
*T* (3)

In the absence of external stress, the deformation is not caused by any mechanical forces. Instead, the deformation produced by the inverse piezoelectric effect is caused solely by the applied electric field, as shown in Equation (4).

*S* = *d*
*^t^E* (4)

The macroscopic piezoelectric properties of composites are determined by their complex internal microstructure. A rational design of the structure can maximize the efficiency of stress transfer and charge accumulation. Meanwhile, systematic optimization of the interfacial microstructure to improve the bonding quality helps reduce charge recombination and improve the uniformity of the polarization electric field. These improvements synergistically enhance the stability and sensitivity of the direct piezoelectric output and the inverse piezoelectric response.

### 2.2. Effect of Microstructural Control on Piezoelectric Behavior

When the piezoelectric phase is randomly and disorderly dispersed within the matrix, the electromechanical conversion efficiency of the material is limited. The large difference in elastic modulus between the piezoelectric phase and the matrix causes the applied mechanical stress to be borne mainly by the matrix. Consequently, stress is not effectively transferred to the piezoelectric phase, resulting in an effective piezoelectric constant (*d*) that is much lower than its theoretical intrinsic value. Additionally, the crystal orientations of individual piezoelectric grains or domains are completely random, leading to disordered polarization directions, as shown in Figure 2b. When mechanical stress is applied, the electric dipole moments generated by differently oriented grains point in different directions. These moments cancel each other out at the macroscopic level, leading to a weak net piezoelectric response or even the complete disappearance of the piezoelectric effect. Consequently, effective charge output cannot be achieved.

An aligned microstructure can significantly optimize the force–electric coupling efficiency. This improvement mainly involves three aspects: (1) Matching the stress field with the electric field. A highly oriented structure provides an ideal continuous pathway for stress transmission. This ensures that mechanical energy is precisely transmitted along the polarization axis, fundamentally preventing stress dispersion and dissipation and thereby maximizing the piezoelectric conversion efficiency [30]. Zeng et al. [31] used computer-aided topological optimization to design the layout of porous PZT. They also fabricated EGaIn liquid-metal circuits by direct writing. The electrode pattern matches the topologically optimized piezoelectric array precisely. This ensures full contact with the PZT. As a result, this configuration forms continuous conductive pathways, eliminates strain dead zones, and maximizes the efficiency of stress transfer to the ceramic. (2) Establishing efficient stress/strain transmission pathways. An aligned arrangement of dipoles enables electric domains to undergo synergistic reorientation and domain-wall migration under external forces. This effectively suppresses internal cancellation and optimizes stress transfer and charge transport pathways. As a result, both electromechanical output and signal stability are significantly improved [30], as shown in Figure 2b [32]. Yuan et al. [32] created a dual mechanically oriented stress field through high-energy ball milling and 3D printing. This induced PVDF molecular chains and PZT nanoparticles to align along the direction of the stress field, forming a highly aligned microstructure and creating efficient stress/strain transfer pathways. The resulting piezoelectric coefficient d_33_ was comparable to that of conventional polarized PVDF films. (3) Reducing dielectric loss and enhancing the effective piezoelectric coefficient. The aligned structure reduces interfacial polarization and leakage current by means of an oriented electric field. At the same time, it synergistically drives the piezoelectric phase. By suppressing ineffective deformation in the polymer matrix, it achieves high electromechanical conversion efficiency and low dielectric loss [33,34,35]. Qi et al. [36] used thermal stretching to prepare a one-dimensional, aligned, and oriented CaCu_3_Ti_4_O_12_ (CCTO) nanowire/graphene/PVDF ternary composite. Thermal stretching induces an oriented alignment and forms numerous layered structures that disrupt conductive pathways, suppress leakage current, and reduce both system polarization and interfacial polarization. This effectively lowers the dielectric loss. At 1 kHz, the 20% CCTO/2% graphene/PVDF oriented composite exhibited a dielectric constant of 22.33 and a dielectric loss of 0.0352, outperforming the random composite.

Therefore, controlling the orientation of the piezoelectric phase and the polymer matrix to create a highly aligned microstructure can directly enhance the material’s intrinsic polarization strength. This represents a key strategy for overcoming the performance limitations of traditional composite materials.

### 2.3. Effect of Interfacial Structure on Piezoelectric Behavior

As the bridge between the two phases of a composite material, the structure and chemical state of the interface determine its overall performance. Interface defects arise from geometric mismatches between the two phases, leading to poor contact and weak bonding. This causes localized stress distortions, disrupts the continuity of electric domains, and hinders stress transfer and charge transport. These defects are a key factor limiting the piezoelectric properties of composite materials. They mainly manifest in the following three aspects. (1) Stress transfer efficiency. The interface serves as a bridge for stress/strain transfer. Differences in elastic modulus between phases or grains can generate stress concentrations or confinement effects at the interface. Using interfacial stress to induce local phase transitions, such as from trigonal to tetragonal, can significantly enhance piezoelectricity. This is commonly observed in relaxor ferroelectric single crystals and ceramics. However, excessive mechanical confinement can suppress the overall strain output. Tang et al. [37] introduced a transition layer of carbon nanotube-containing relaxor ferroelectric terpolymer at the interface between the three-dimensional PZT ceramic scaffold and the PDMS elastomer. This layer established pathways for polarization and mechanical load transfer, alleviated dielectric and mechanical mismatches between the two phases, and suppressed localized electric field concentration. As a result, the composite achieved a piezoelectric charge coefficient of 120 pC/N and an electromechanical coupling coefficient of 65%, while maintaining high flexibility and cycling stability under a large strain of 50%. (2) Electric field distribution and polarization efficiency. The interfacial electric field arises from electron migration and polarization effects. A well-designed interface not only induces localized polarization and forms an interfacial dipole layer but also maximizes the interfacial surface area. This enhances the piezoelectric response. At the same time, it reduces defects, suppresses charge leakage, and ensures the stability of the piezoelectric output [38,39]. Liu et al. [40] modified BTO nanoparticles with DET, which promoted the uniform dispersion of BTO within the PVDF matrix and improved the compatibility and interfacial polarization between the nanoparticles and the polymer matrix. The d_33_ value of the 3 wt% DET–BTO/PVDF composite increased from 12.6 pC/N for pure PVDF to 40.3 pC/N. Its remanent polarization was approximately 2.5 times that of BTO/PVDF. The dielectric constant reached its maximum at a filler content of 3 wt%, while the dielectric loss remained below 0.04 in the frequency range of 0.1–1000 kHz. This indicates that the enhanced interfacial polarization was not accompanied by obvious deterioration of electric current or dielectric loss. (3) Dielectric and mechanical losses. The interface enables continuous stress transfer, reducing chain slippage, delamination, and friction, thereby lowering mechanical losses. A strong interface can buffer differences in elastic modulus, preventing plastic dissipation caused by stress concentration. It can also suppress the formation and propagation of microcracks, reducing friction and additional losses [30,41,42]. Su et al. [29] formed strong hydrogen bonds between the –OH end groups on the surface of Ti_3_C_2_T_x_ nanosheets and the C–H and C–F groups of PVDF molecules. This forced the PVDF molecular chains to transform into the β phase, resulting in an aligned arrangement of dipoles. It also reduced defects and carrier pathways, suppressed conductance loss and interfacial polarization loss, and lowered the dielectric loss tangent (tanδ), as shown in Figure 2c.

## 3. Aligned Microstructure for Enhanced Piezoelectric Behavior

**Figure 3:**
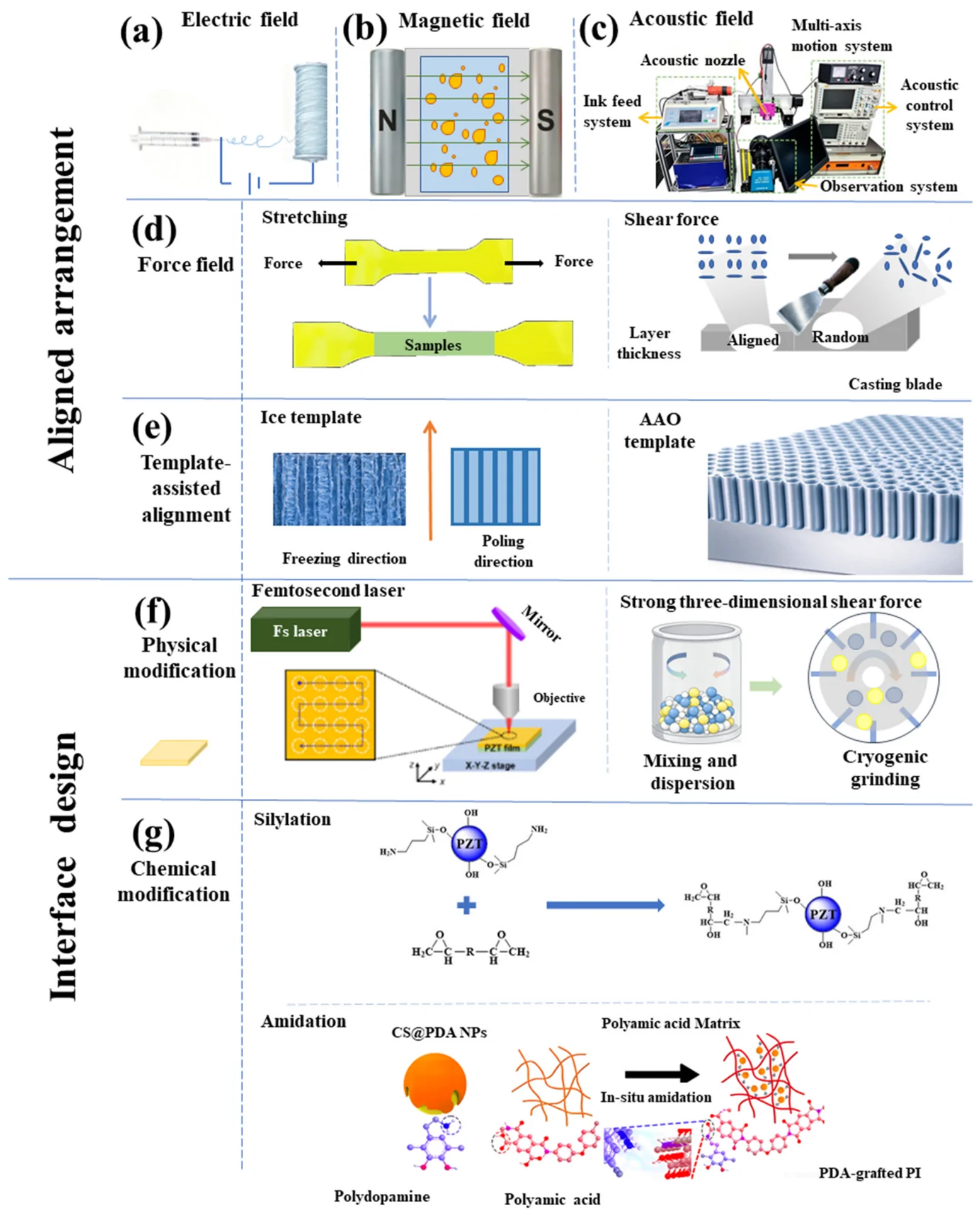
Methods for aligned microstructure of piezoelectric composites: (**a**) electric field; (**b**) magnetic field; (**c**) acoustic field [43], © 2026 by the authors. Licensee MDPI, Basel, Switzerland; (**d**) force field; (**e**) template-assisted alignment; (**f**) physical modification [44], © 2025 by the authors. Licensee MDPI, Basel, Switzerland; (**g**) chemical modification [45,46], silylation [46], © 2014 Society of Plastics Engineers; amidation [45], © 2026 The Author(s). Advanced Functional Materials published by Wiley-VCH GmbH.

The aforementioned piezoelectric mechanisms indicate that the efficiency of charge accumulation and migration within the material directly affects the intensity of the piezoelectric response. The microstructure of piezoelectric composites varies depending on factors such as the preparation process and the types of chemical bonds between the two phases; their core characteristics are primarily reflected in their composition and interfacial structure. The efficiency of stress transfer and the uniformity of the electric field distribution depend on the bonding state and arrangement of the two phases at the interface. Therefore, achieving control over the microstructure by regulating composition and interfacial structure is essential for enhancing the piezoelectric performance of composite materials; the primary approaches are shown in Figure 3. The characteristics of aligned microstructure for piezoelectric composites are shown in Table 1.

### 3.1. Field-Assisted Alignment

#### 3.1.1. Electric Field

- Electrospinning

**Figure 4:**
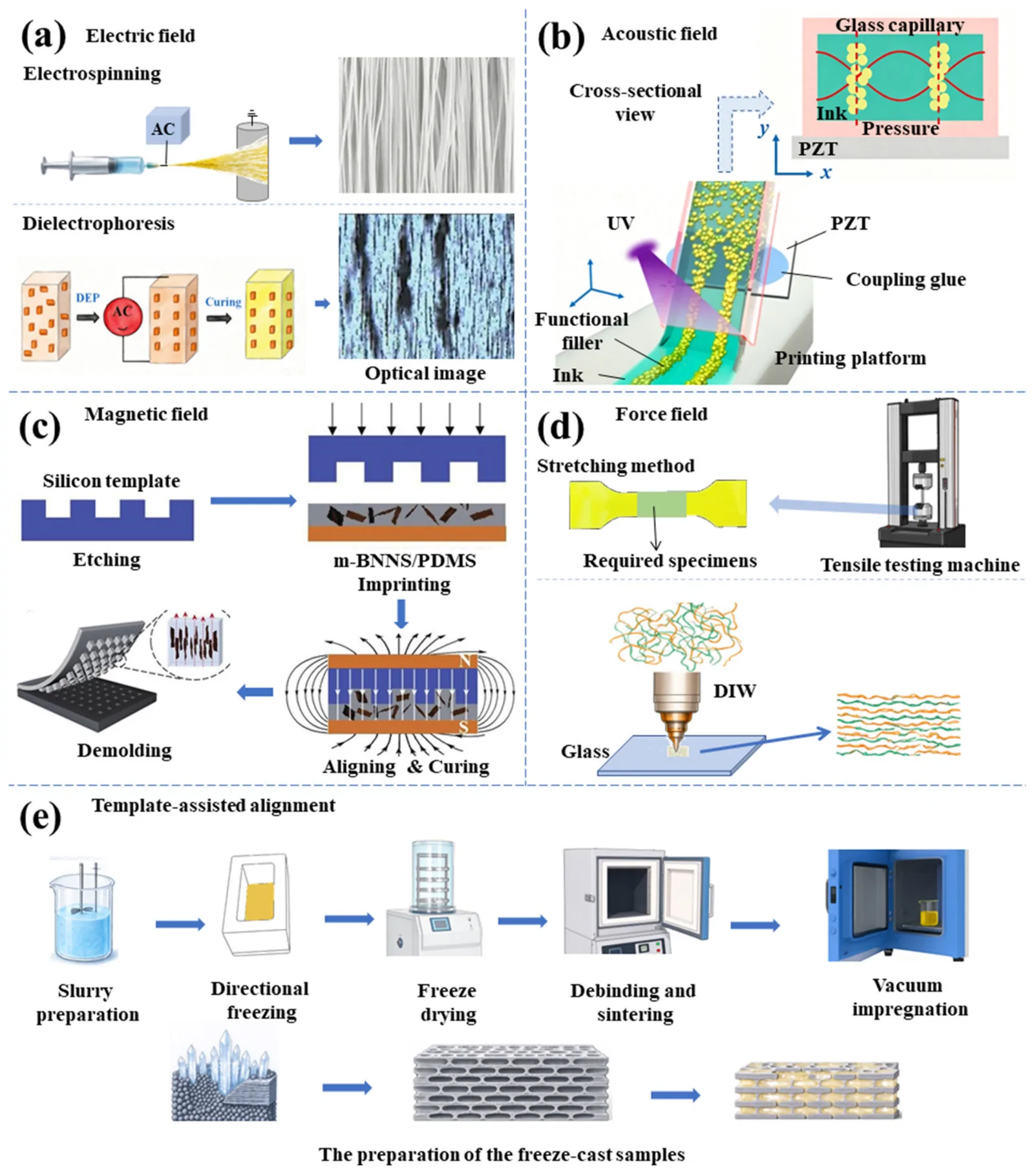
Field-assisted alignment includes: (**a**) electric field [49,62], © 2020 Wiley-VCH GmbH; (**b**) acoustic field [43], © 2026 by the authors. Licensee MDPI, Basel, Switzerland; (**c**) magnetic field [56], © The Author(s) 2024. Published by Science Press and EDP Sciences; (**d**) force field; (**e**) template-assisted alignment.

Electrospinning is a novel technology for preparing nanomaterials. It uses a high-voltage electric field to induce a precursor droplet to form a Taylor cone and be ejected as a jet, thereby producing nanofibers, as shown in Figure 4a. This technology requires simple equipment and can produce fibers with controllable diameters. However, it is sensitive to process parameters and has low production efficiency, thereby limiting scalability. The final morphology and structure of the fibers are influenced by two types of factors. The first type is process parameters, including voltage, solution feed rate, ambient temperature, humidity, and collection distance. Excessively high voltage can cause fiber thickening or breakage due to jet instability. If the collection distance is too short, insufficient stretching and incomplete solvent evaporation occur; if it is too long, the fiber diameter increases and the piezoelectric performance decreases. A solution feed rate that is too low tends to cause beading, whereas a rate that is too high leads to droplet coalescence, fiber adhesion, or entanglement [47]. The second type is the properties of the spinning solution itself, covering key indicators such as polymer molecular weight and solution concentration. An increase in molecular weight can lead to larger fiber diameters and a more regular structure. It also promotes molecular chain orientation, enhances crystallinity, and increases the β-phase content. This results in a simultaneous improvement in both mechanical properties and piezoelectric output. For example, Zaarour et al. [48] prepared an oriented PVDF fiber membrane and used it as the active layer in a piezoelectric nanogenerator (PENG). At an impact frequency of 5 Hz, the device produced a voltage of 2.8 V and a current of 4 µA. If the solution concentration is too low, beading and spindle-shaped structures are likely to form. Conversely, a concentration that is too high can lead to needle clogging and an increase in fiber diameter.

- Dielectrophoresis

Dielectrophoresis (DEP) is a phenomenon in which an alternating electric field induces electric dipoles in piezoelectric powder/polymer slurries. Driven by Coulomb forces, the particles assemble into long-chain structures aligned along the field direction. After the slurry cures, this oriented filler structure is retained within the matrix [50], as illustrated in Figure 4a. DEP is simple to operate and allows the direction of movement to be switched by varying the electric field frequency. However, it is effective only in non-uniform electric fields. Its effectiveness is primarily governed by a combination of factors, including particle characteristics (such as dielectric properties, size, and shape), liquid characteristics (such as conductivity, viscosity, and dielectric constant), electric field strength and frequency, and electrode spacing [51]. For example, Yildirim et al. [49] used dielectrophoresis to align lead zirconate titanate (PZT) particles with graphene nanoplates (GNPs). They formed 1-3 piezocomposites by constructing vertically aligned nanocolumns in a polydimethylsiloxane (PDMS) matrix, as shown in Figure 4a. The study found that the d_33_ coefficient increased, with the g_33_ coefficient reaching up to 0.709 Vm/N. The relative permittivity (ε) increased with rising concentration. The peak power density reached 2.5 mW/cm^3^. A higher electric field strength produces a stronger driving force, which leads to faster particle orientation, longer chains, and more aligned arrangements. Low frequencies facilitate the polarization orientation of PZT particles. In contrast, high frequencies prevent dipoles from keeping pace with electric field reversals, thereby weakening both polarization and the driving force. The best orientation results are achieved at particle concentrations between 0.25 and 1 vol%. At higher concentrations, excessive crowding of particles results in a generally random distribution.

#### 3.1.2. Acoustic Field

Acoustic field induction uses physical effects generated by sound waves within materials to directionally align piezoelectric phases inside a polymer matrix. These effects, including acoustic radiation force, acoustic streaming, and cavitation, enable the construction of aligned microstructures. This technique offers strong penetration capability, controllable orientation, and broad applicability. However, sound waves attenuate during propagation, and the fabrication process is complex. The key influencing parameters include the size and shape of the piezoelectric filler, the viscosity of the matrix, and process parameters such as ultrasonic frequency and amplitude [53]. Lu et al. [54] proposed an acoustically assisted projection stereolithography (A-PSL) process. This method uses the acoustic swimming effect. Piezoelectric elements at the bottom of the resin tank generate a controllable standing wave acoustic field. This field drives the precise, oriented alignment and diverse patterning of functional phase particles within the liquid resin under the influence of acoustic radiation forces and drag forces. The particles used include tungsten, aluminum, titanium, and copper, with sizes ranging from 70 nm to 75 µm. The size and shape of the fillers determine their force response and arrangement patterns within the acoustic field. For example, unidirectional parallel line arrangements are achieved using plate-shaped piezoelectric elements. Meanwhile, single-layer partitioned orthogonal orientations and multi-layer orthogonal orientations can be achieved through time division or zone division control of these elements. Ultrasonic frequency and amplitude directly regulate the pressure distribution in the acoustic field and the magnitude of the acoustic radiation force. These, in turn, determine the orientation, uniformity, and pattern structure of the fillers. The lower the matrix viscosity, the smaller the Stokes drag force, making the particles easier to move and the orientation precision more controllable.

#### 3.1.3. Magnetic Field

The magnetic field-induced method uses an external magnetic field to generate magnetic dipole forces that drive functional phase particles to align with the field direction, forming an aligned structure. This technique can maintain high performance at low filler content and offers good process controllability. However, a drawback is that it usually requires adding magnetic particles to enhance magnetic responsiveness, which increases process complexity. The effectiveness of magnetic field-induced orientation depends on multiple factors, primarily including magnetic field parameters, filler properties, matrix properties, and process conditions [55,57]. Xin et al. [56] combined magnetic field alignment with nanoimprinting technology. By magnetically modifying boron nitride nanosheets (BNNS), they achieved vertical alignment of the filler within the matrix under an external magnetic field, producing magnetic boron nitride nanosheet/polydimethylsiloxane (m-BNNS/PDMS) microcolumn array piezoelectric sensors, as shown in Figure 4c. Their study found that a strong magnetic field can drive the magnetically modified BNNS to overcome the viscous resistance of the matrix, enabling orderly vertical alignment along the magnetic field lines. This significantly enhanced the piezoelectric response, and the piezoelectric output was twice that of randomly dispersed thin film samples. The morphology, structure, and magnetic properties of BNNS directly determine its magnetic field responsiveness. The size, state, and loading of the filler affect the uniformity of alignment, and excessive loading can easily cause agglomeration and compromise the alignment effect. The lower the viscosity of PDMS and the slower its curing rate, the more favorable the conditions for full BNNS alignment.

#### 3.1.4. Force Field

The force field induction method applies a stress field, such as shear forces or tensile forces, to align particles or chain segments along the direction of the force. This method is simple, controllable in orientation, and highly adaptable. However, its drawbacks include a single orientation direction and a tendency to produce residual stresses.

- Stretching Method

This forces molecular chains or units to orient, align, and fix themselves along the direction of stretching, creating an aligned structure. Mohammadi et al. [63] controlled the content and orientation of the polar β phase in PVDF by stretching, which in turn determined its piezoelectric performance, as shown in Figure 4d. During uniaxial stretching at 87 °C, raising the stretching rate from 10 to 50 cm/min reduced stress relaxation, promoted the α-to-β phase transition, and improved the orientation of β crystallites. As a result, the β-phase content increased from 77.6% to 83.6%. Increasing the stretch ratio from 1 to 6.5 further raised the β-phase content, with a maximum of 86.5%. Together, these effects increased the d_33_ value after poling from 0 pC/N to 33 pC/N, indicating that the combination of a high stretching rate and a high stretch ratio is key to achieving high piezoelectric performance.

- Shear force

Shear-induced orientation is a process in which the shear force generated by relative motion between fluid layers aligns initially disordered units, such as molecular chains, nanoplates, and fibers, along the shear flow direction. Common 3D-printing processes and vat photopolymerization (VPP) technology both involve shear forces to achieve oriented arrangement of materials [59]. Li et al. [64] fabricated MXene/P(VDF-TrFE) composite piezoelectric films via extrusion-based direct ink writing (DIW)-based 3D printing. During printing, the shear stress inside the nozzle aligned the polymer molecular chains along the printing direction. This alignment, together with the hydrogen bonding between the functional groups on the MXene surface and the P(VDF-TrFE) chains, anchored the dipoles effectively without any additional poling treatment. As a result, the piezoelectric performance of the composites was greatly improved. The optimized piezoelectric nanogenerator delivered an open-circuit voltage of 5.5 V, a short-circuit current of 1.1 µA, and a maximum output power density of 68 μW/cm^3^ under a cyclic pressure of 22 N at 2 Hz.

### 3.2. Template-Assisted Alignment

Template-directed alignment uses preset microscale and nanoscale templates to guide molecular chains within a material into alignment under template confinement, with the assistance of an external field. The template method offers advantages such as low cost, high flexibility, and good structural controllability. Key factors influencing the preparation of micromaterials and nanomaterials via the template method include the pore size, orientation, and surface functional groups of the template, the properties of the precursor solution, and process parameters. These factors significantly modulate the morphology and microscopic interfacial state of the material, thereby determining its macroscopic properties, such as piezoelectricity [60,61]. Ceramics grown using the template method exhibit a piezoelectric response 2 to 3 times higher than that of randomly grown ceramics, making this method one of the effective approaches for enhancing ferroelectric piezoelectric performance. Zhang et al. [65] used freeze casting, also known as the ice template method, with water as the freezing medium, as shown in Figure 3e. Unidirectional freezing allowed ice crystals to grow in an oriented manner. After sintering, the ice sublimated, leaving behind oriented layered pores and simultaneously forming ceramic bridges. The study found that as porosity increased, the dielectric constant decreased, and the piezoelectric and pyroelectric energy harvesting values significantly exceeded those of dense PZT. Chen et al. [58] used the AAO template electrohydrodynamic deformation method to achieve the vertically aligned growth and in-situ polarization of P(VDF-TrFE) nanowires in a single step, as shown in Figure 3e. The assembly was then thermally maintained at 160 °C, which is higher than the melting temperature of P(VDF-TrFE) (T_m_ = 150 °C), while a DC voltage of 500 V was applied between the template and the substrate. The electric field induced the vertical alignment of the molecular dipoles, achieving in situ polarization. After cooling, the template was removed, yielding a vertically aligned nanowire array with an output voltage of approximately 4.8 V and a current density of 0.11 µA/cm^2^.

To allow a clearer comparison, a summary is given at the end of this section, together with the case studies in Section 3. For microstructure design, the comparison covers d_33_, g_33_, piezoelectric output, stability, and other properties.

Table 1 and Table 2 summarize various alignment strategies. However, direct comparison of absolute values is difficult because the literature uses different reference baselines and testing conditions. Each strategy has its own trade-offs and limitations. Electric-field-assisted alignment can effectively improve the piezoelectric coefficient, but it requires high voltage. Magnetic-field-assisted alignment needs magnetic fillers, which tend to increase dielectric loss and reduce the effective piezoelectric response. Force-field-assisted alignment gives a large output enhancement, but it depends heavily on the loading mode and the viscoelasticity of the material. Template-assisted alignment provides only limited improvement and involves complex fabrication. Acoustic-field-assisted alignment is still relatively understudied, and reported piezoelectric data are scarce. Overall, field-assisted alignment can greatly improve the performance of piezoelectric composites. Therefore, choosing an alignment scheme requires a balance among the magnitude of performance enhancement, filler composition, fabrication complexity, and engineering scalability.

## 4. Interface Design for Enhanced Piezoelectric Behavior

Piezoelectric composites combine the flexibility of polymers with the piezoelectric properties of ceramics, offering broad application prospects. However, these materials still face challenges, including uneven dispersion of ceramic particles within the matrix and weak interfacial bonding between the ceramic and polymer phases, which severely limit their piezoelectric performance and stability. To address these issues, researchers have explored two major categories of interfacial modification strategies: physical modification and chemical modification. These strategies aim to optimize the two-phase interface, enable precise design and control of the interfacial structure, and ultimately enhance piezoelectric performance. The characteristics of interface design for piezoelectric composites are shown in Table 3.

### 4.1. Physical Modification

Physical modification alters the microstructure of an interface through physical means, such as mechanical force, heat treatment, or physical coating, without changing the chemical composition or forming new chemical bonds. To a certain extent, this can enhance physical adsorption at the interface. This method is simple and relatively low in cost. However, the improvement in interfacial bonding strength is limited, and the modification effects are not long-lasting.

**Figure 5:**
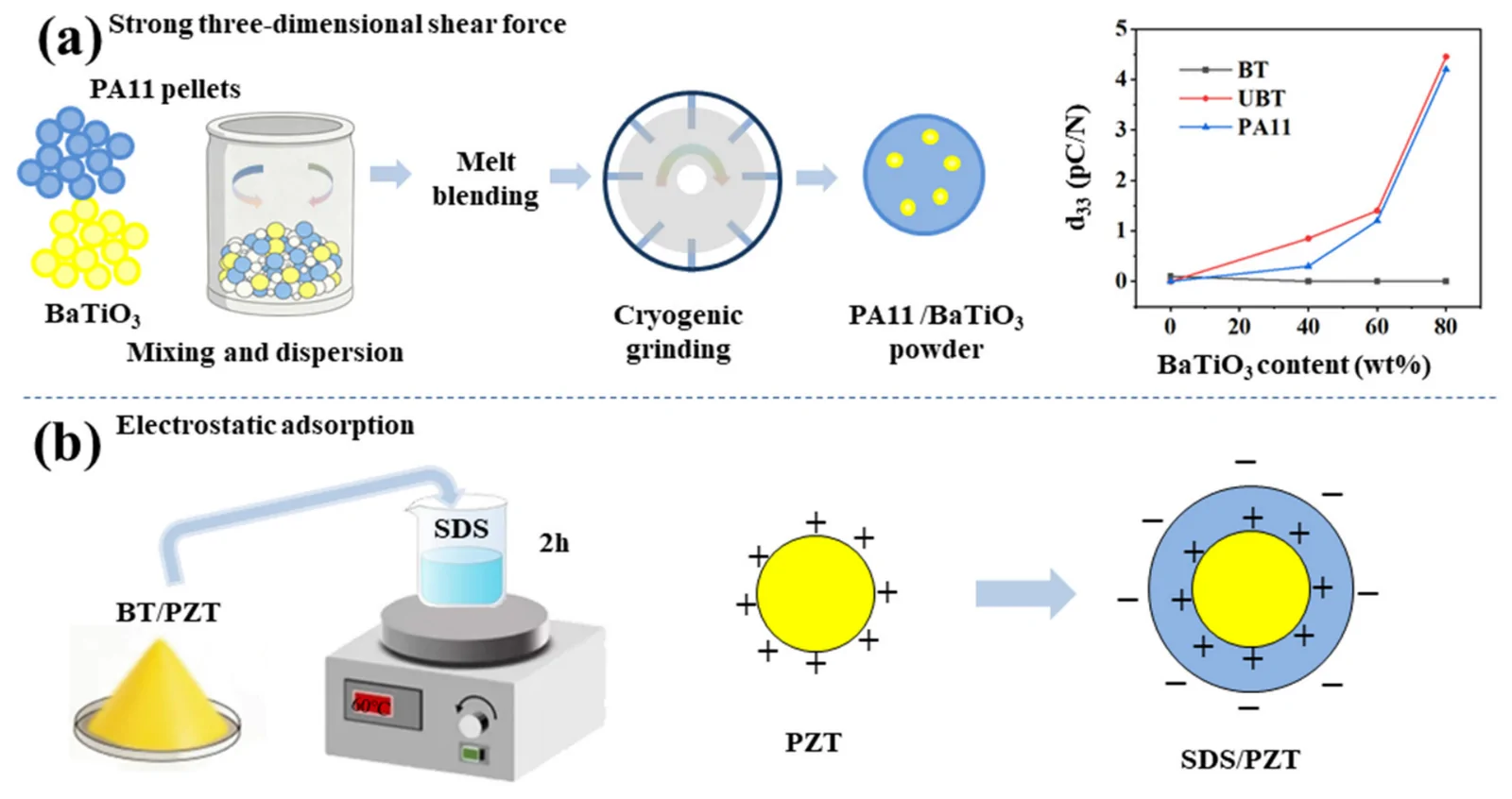
Physical modification includes: (**a**) strong three-dimensional shear force [80]; (**b**) electrostatic adsorption [79].

Qi et al. [80] proposed a novel physical modification method that combines solid-state shear milling, melt blending, and low-temperature grinding. By applying strong three-dimensional shear forces, they achieved uniform dispersion of BaTiO_3_ nanoparticles in a PA11 matrix. Finally, PA11/BaTiO_3_ piezoelectric nanocomposites were fabricated via selective laser sintering (SLS), as shown in Figure 5a. The solid-state shearing disrupted the BaTiO_3_ nanoparticles and broke the PA11 molecular chains, thereby increasing the contact area and the number of interfacial bonding sites. This effectively enhanced the interfacial stress transfer efficiency of the composite and significantly reduced energy loss during mechanical-to-electrical energy conversion. The d_33_ and g_33_ values of the BT80 sample were 47 times and 8 times those of pure PA11, respectively.

Mahato et al. [79] utilized the anionic surfactant sodium dodecyl sulfate (SDS) for the physical surface modification of BT and PZT ceramic particles, as shown in Figure 5b. SDS adsorption induced a negative surface charge, which enhanced electrostatic repulsion, thereby mitigating particle agglomeration and ensuring uniform dispersion in the PVDF matrix. These charged surfaces subsequently served as nucleation sites, interacting electrostatically with the partially positive −CH_2_^+^ moieties of PVDF to induce the TTTT configuration. Consequently, the β-phase fractions reached 71.85% for BT-PVDF and 54% for PZT-PVDF. These findings highlight that physically adsorbed surfactants can indirectly govern the polar phase content by tuning particle dispersibility and interfacial charge, presenting a viable pathway for interfacial modification independent of chemical bonding.

### 4.2. Chemical Modification

Unlike physical modification methods, chemical modification improves interfacial compatibility at the molecular level through chemical bonding, surface functional group modification, and molecular grafting. It offers advantages such as simple preparation, low cost, and good durability [81,82]. Common chemical modification methods include silylation, amidation, and grafting.

#### 4.2.1. Silylation

Silane coupling agents are classic reagents for the chemical modification of piezoelectric composites. These are compounds with bifunctional structures, such as γ-glycidyl ether-oxypropyltrimethoxysilane (KH560), 3-aminopropyl trimethoxysilane (APS), and 3-(methacryloyloxy)propyl trimethoxysilane (TMSPM). The functional group at one end can form a covalent bond with active groups on the ceramic surface, such as -OH, while the functional group at the other end can cross-link with the polymer matrix [71].

Wu et al. [71]. investigated 1-3 type helical PZT/epoxy composites. They immersed the PZT in solutions of the silane coupling agent KH560 (pH = 4) and the titanate coupling agent NDZ201 to ensure thorough interaction between the coupling agents and the ceramic. The results showed that the d_33_ (546 pC/N) and hydrostatic pressure Figure of merit (FOM) (13.4 × 10^−12^ m^2^/N) of the KH560-modified composite were 7.5% and 29.5% higher, respectively, than those of the unmodified samples. In contrast, NDZ201 modification led to a decline in performance. The primary reason is that KH560 hydrolyzes in an acidic aqueous solution at pH 4 to form silanol groups, which then undergo dehydration condensation with hydroxyl groups on the ceramic surface, thereby achieving chemical grafting. Furthermore, intermolecular condensation forms a stable -Si-O-Si- three-dimensional cross-linked network. NDZ201 chemically absorbs the hydroxyl groups on the ceramic surface via titanium–oxygen and phosphorus–oxygen bonds. However, due to its long molecular chains and numerous functional sites, molecular entanglement is likely to occur, and the reaction is not fully carried out because of steric hindrance.

**Figure 6:**
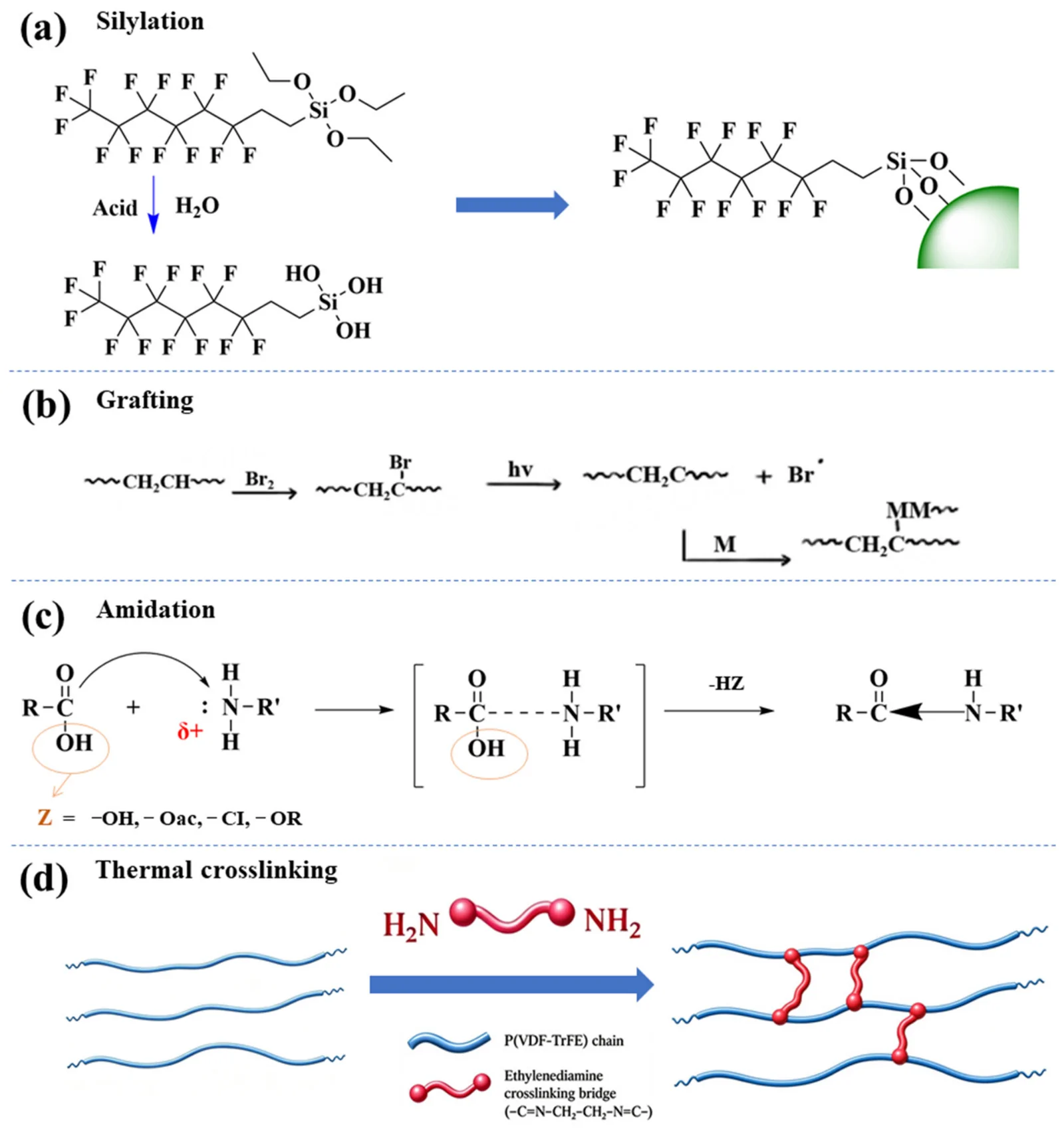
Chemical modification includes: (**a**) silylation [72], © 2021 The Author(s). Published by the Royal Society of Chemistry; (**b**) grafting [82], © The Author(s) 2023; (**c**) amidation [82], © The Author(s) 2023; (**d**) other chemical modifications [83].

Modification with silane coupling agents enables PVDF to possess both higher β-phase content and electrical activity. Li et al. [72] used 1H, 1H, 2H, 2H-perfluorodecyltriethoxysilane (PFDTES) to chemically modify the hydrophobic surfaces of barium titanate (BTO) nanoparticles, thereby preparing FD-BTO/PVDF nanocomposite films, as shown in Figure 6a. The study found that the piezoelectric coefficient d_33_ increased with rising FD-BTO content, reaching 69.1 pC/N at 50 wt%. This is because the hydrophobically modified FD-BTO, acting as a nucleating agent, significantly increased the content of the piezoactive β phase in PVDF and formed strong hydrogen bonds with PVDF. Meanwhile, the hydrophobic modification by PFDTES reduced the surface energy of BTO, enabling uniform dispersion of the filler and eliminating interfacial defects.

Modifications using other silane coupling agents have shown similar effects, promoting nanoparticle dispersion and enhancing interfacial interactions. Jin et al. [73] surface-modified PZT nanoparticles using TMSPM (3-(trimethoxysilyl)propyl methacrylate) and employed photopolymerization (3D printing) technology to fabricate a novel flexible piezoelectric sensor. By utilizing the condensation of the trimethoxysilyl group with hydroxyl groups on the PZT surface and the radical polymerization of the methacryloyloxy group with the photosensitive resin, a chemical bridge was formed between the PZT and the organic matrix. The study found that the sensor achieved a piezoelectric constant of 25.8 pC/N, with an output voltage 1.67 times higher than that of traditional PVDF sensors, a sensitivity of 1.19 V/N, and a response time of only 58 ms. Nguyen et al. [46] ultrasonically modified PZT nanoparticles using an aqueous solution of γ-amino propyl trimethoxy silane (γ-APS), then compounded them with epoxy resin and prepared the composite material via cast film formation. The study found that FT-IR confirmed the formation of Si-O-PZT bonds. PZT was uniformly dispersed in the epoxy resin matrix without significant agglomeration, and γ-APS effectively improved the interfacial bonding between the filler and the matrix. At 30 °C and 1 kHz, the dielectric constant increased by 20–40%, achieving simultaneous optimization of dielectric and thermal properties at low PZT content.

#### 4.2.2. Grafting

Through reactions such as free-radical and ring-opening polymerizations, polymer segments identical to or compatible with the matrix polymer chains are grafted onto the ceramic surface. This causes the modified ceramic to form chain entanglement and cross-linking with the matrix, thereby improving nanoparticle dispersion, as shown in Figure 6b. Graft modification offers advantages such as mild reaction conditions, simple operation, and cost-effectiveness [74,75,84].

Chen et al. [75] modified PZT via chemical grafting with tetradecylphosphonic acid (TDPA), incorporating a hydrophobic long-chain alkane structure compatible with hydrophobic PVDF into the PZT. The modification increased steric hindrance, hindering the mutual attraction of PZT particles and improving the dispersion of PZT in PVDF. The P-OH groups at the ends of TDPA formed P-O-M bonds with the positively charged metal ions on the PZT surface, enhancing the interfacial bonding strength. The study found that when 10 wt% m-PZT was added, the piezoelectric constant of the composite film reached 27.1 pC/N and remained at 26.8 pC/N after 2000 bending cycles. Under a cyclic pressure of 290 N, the output voltage reached up to 3.426 V, significantly outperforming the unmodified PZT composite film (1.994 V) and the pure P(VDF-TrFE) film (1.958 V). Shi et al. [76] employed a covalently bonded surface grafting method achieved via surface-initiated in-situ atom transfer radical polymerization (ATRP) to modify BaTiO_3_ nanowires, depositing a PMMA layer 8–10 nm thick on the surface of the BT NWs. After modification, the output power of the piezoelectric composite was 2.2 times that of the unmodified sample and 7.6 times that of the pure P(VDF-TrFE) -based sample. This is because the PMMA layer reduces the surface energy of the BaTiO_3_ nanowires, preventing significant agglomeration and avoiding piezoelectric dipole cancellation. Furthermore, the PMMA layer forms an interfacial bond between the BaTiO_3_ nanowires and the P(VDF-TrFE). The high modulus of PMMA allows mechanical strain applied to the matrix to be transmitted more efficiently to the surface of the BaTiO_3_ nanowires.

#### 4.2.3. Amidation

An amide formation reaction essentially involves an amine acting as a nucleophile to attack the highly electron-deficient carbonyl carbon in a carboxylic acid derivative, such as an acyl chloride, an acid anhydride, or an activated ester. This attack forms a negatively charged tetrahedral intermediate. Subsequently, an elimination reaction removes the leaving group, such as a chloride ion or a carboxylate anion, thereby reestablishing the carbonyl structure and forming a stable amide bond, as shown in Figure 6c. The advantages of amide modification include mild reaction conditions, good stability, and broad applicability. The disadvantages include cumbersome post-treatment and relatively slow reaction rates [77].

The covalent bonds formed by amidation can act as highly efficient “molecular bridges”, improving interfacial bonding and thereby enhancing the electrical properties of the composite. Li et al. [78] systematically improved the piezoelectric properties of P(VDF-TrFE) composite films through dual amidation modification. First, carbon-based nanomaterials were subjected to amide modification to introduce hydrogen-bonding sites and induce the formation of the β-phase. Second, the polymer dispersant was prepared via an amidation reaction using styrene-maleic anhydride copolymer (SMAz) and polyether monoamine (M1000) as raw materials. This reaction converted the anhydride groups into amide bonds, yielding the dispersant SMAz-M, which improved dispersion and enhanced hydrogen bonding. These modifications increased the β-phase content of the modified composite film to 86.4% and raised the piezoelectric constant to 26.4 pC/N. Lee et al. [77] compounded amine-functionalized PZT nanoparticles (PZT-NH_2_ NPs) with a thermoplastic triblock copolymer without the need for additional dispersion enhancers. The amine-modified PZT forms covalent bonds with maleic anhydride molecules in the polymer matrix, resulting in the uniform distribution of PZT-NH_2_ NPs within the matrix and a significant improvement in piezoelectric performance.

#### 4.2.4. Other Chemical Modifications

In addition to surface modification, direct chemical regulation of the polymer’s main chain can also enhance material performance, such as by modifying the main chain or by constructing intermolecular cross-linking networks. Yuan et al. [85] used strong alkali (NaOH) to remove hydrogen fluoride, introducing C=C double bonds into the P(VDF-TrFE) main chain. This modulated the energy competition between the ordered all-trans and disordered 3/1-helical crystalline conformations, increasing the piezoelectric coefficient d_33_ to −90.5 pC/N, which is three times that of pure PVDF. It also significantly increased the dielectric constant and yielded excellent mechanical properties and processability. This method does not require complex copolymerization, strong electric fields, or harsh processing conditions, and it enables solution processing and large-scale fabrication of flexible piezoelectric films. Previous studies have suggested that crosslinking modification disrupts crystallization and reduces piezoelectric performance, or that the use of high-volume crosslinking agents, such as polyethylene glycol, results in significant steric hindrance. This causes crosslinking to occur only in amorphous regions, leading to weak conformational heterogeneity. Yuan et al. [83] proposed a new strategy for intermolecular crosslinking, using small-molecule crosslinking agents such as ethylenediamine and 1, 2-propanediamine to crosslink P(VDF-TrFE) via spin-coating and thermal crosslinking, as shown in Figure 6d. Experimental and theoretical results confirm that small molecule crosslinking agents can generate strong local conformational heterogeneity near the crosslinking sites, where even a small electric field can readily induce large polarization and strain, thereby increasing d_33_. The d_33_ value for the ethylenediamine-crosslinked sample is approximately −95.0 pC/N, and that for the 1, 2-propylenediamine-crosslinked sample is approximately −93.4 pC/N. Crystallinity remains unchanged after crosslinking, and the samples exhibit good flexibility and stability.

In addition, core–shell structures can be formed to coat PZT using a chemical precipitation method, which is simple, low-cost, and environmentally friendly. Cao et al. [86] used a precipitation reaction between MgCl_2_•6H_2_O and KOH to deposit a layer of insulating magnesium oxide (MgO) on the PZT surface. The hydroxyl groups on the surface can form hydrogen bonds with the F atoms in PVDF, thereby reducing PZT agglomeration. The results show that both the dielectric constant and breakdown strength of the composite material are enhanced, while dielectric loss is reduced. The metallic conductive phase can be deposited onto the surface of the piezoelectric phase as nanoparticles, forming a metal-ceramic chemical bond at the interface and constructing a core–shell structure with functional synergy. Zhou et al. [87] used an in-situ photoreduction method to attach Ag nanoparticles to BCZT nanostructures (BCZTNWs), which were then composited with PVDF to prepare the BCZT@Ag/PVDF piezoelectric composite. Zhou et al. [87] proposed that the Ag-BCZT interface on the upper surface is reverse-biased, making it difficult for free charge carriers to cross it. In contrast, the Ag-BCZT interface on the lower surface is biased forward, allowing free electrons to pass through the interface and migrate toward the Ag nanoparticles. This generates additional polarization charges and localized piezoelectric potentials, which significantly enhance the composite material’s overall macroscopic piezoelectric response.

To achieve a clearer comparison, a summary of interface engineering methods is presented at the end of this section, together with the case studies in Section 4. The comparison covers d_33_, piezoelectric output, and other properties, as shown in Table 4. Together with Table 3 and Table 4, and the case analysis, these results help clarify the structure–property relationship and provide practical guidance for selecting suitable strategies.

Table 3 and Table 4 show that different interfacial design strategies enhance the output performance of piezoelectric composites to very different extents, and that each method has its own trade-offs. Under comparable conditions, that is, the percentage improvement relative to the corresponding unmodified film, amidation and grafting generally give the largest enhancements. Amidation is the preferred strategy when the filler has matching surface functional groups. Grafting is efficient and structurally controllable, but its post-treatment is cumbersome and its reaction rate is slow, which makes scale-up difficult. Silanization is a mature technique and can achieve relatively high piezoelectric enhancement, but it tends to cause filler agglomeration and residual impurities at high filler loadings. Physical modification is simple and low-cost, but its enhancement is relatively limited, and its interfacial stability and durability are insufficient. Overall, choosing an interfacial modification strategy requires a balance among piezoelectric gain, reaction conditions, post-treatment complexity, and long-term interfacial stability.

## 5. Applications

Piezoelectric materials can convert energy from the natural world into electrical energy. They have seen rapid development in recent years and have demonstrated tremendous potential in areas such as sensors and wearable electronics. This section analyzes and summarizes research progress on piezoelectric composites and their applications in various fields.

### 5.1. Smart Sensors

**Figure 7:**
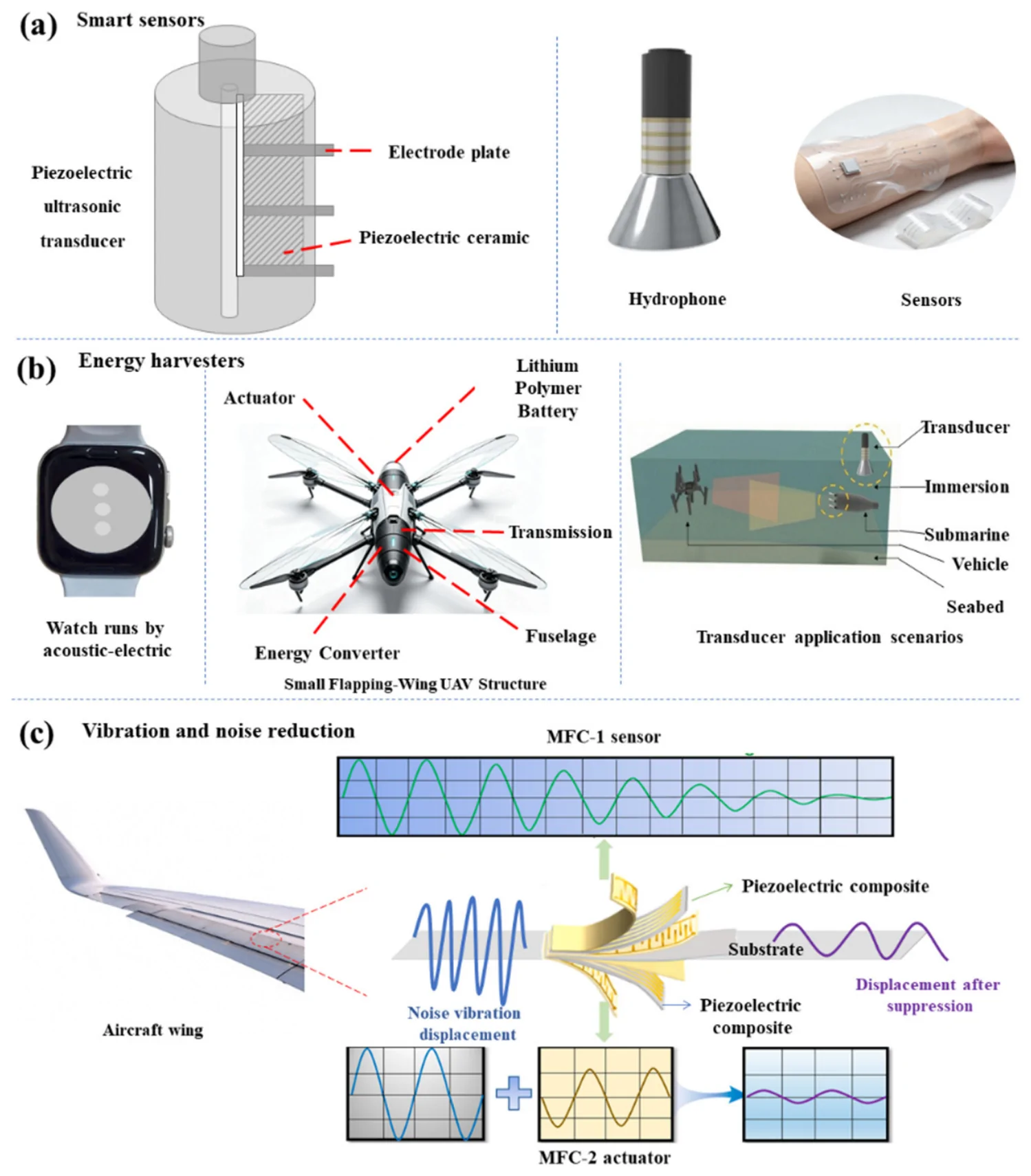
Applications: (**a**) transducer structure and sensors, © 2023 by the authors. Licensee MDPI, Basel, Switzerland; (**b**) energy harvesters [92,93], © 2023 by the authors. Licensee MDPI, Basel, Switzerland; (**c**) vibration and noise reduction [94], © 2026 by the authors. Licensee MDPI, Basel, Switzerland.

Due to their superior performance, piezoelectric materials are the material of choice for human body detection sensors. Traditional piezoelectric polymers have low intrinsic piezoelectric coefficients, while inorganic piezoelectric materials suffer from poor dispersibility, which limits sensor performance [89]. Yang et al. [90] developed a high-performance PDA@BTO/PVDF composite film sensor. The study found that under a 12 N impact, the peak output voltage reached 9.3 V, which is 13.3 times that of pure PVDF, and the response time was only 61 ms. Jadidian et al. [91] conducted research on piezoelectric materials and transducers for ultrasonic testing equipment. They fabricated a single-element ultrasonic transducer with a center frequency of 25 MHz, using lead-free KNN-LT-LS ceramics, 1-3 PZT fiber/epoxy composite, and PVDF film as active elements, as shown in Figure 7a. The study found that the 1-3 PZT fiber/epoxy composite exhibits a high piezoelectric constant of 400 pC/N and a thickness coupling coefficient (k_t_) of 0.64, achieving an ultra-wide −6 dB bandwidth of 118%.

### 5.2. Energy Harvesters

Various embedded sensors in everyday life, such as medical implants, typically rely on rechargeable power sources, while a vast amount of mechanical energy in the environment, such as human movement, remains underutilized, as shown in Figure 7b. Singh et al. [95] incorporated MgO nanoparticles and methyl ethyl ketone (MEK) into a P(VDF-TrFE) copolymer, which self-assembled into a flexible energy harvesting device with a metal-insulator-metal structure. The piezoelectric coefficient increased by approximately 45%, and the output voltage for energy harvesting reached 2 V, more than double that of pure P(VDF-TrFE). Furthermore, the piezoelectric coefficient showed no decay after 10,000 bending cycles, demonstrating that the material possesses high piezoelectric performance, low leakage current, and strong fatigue resistance. Stuber et al. [96] used dielectrophoresis to align lead-free potassium-sodium-lithium niobate (KNLN) ceramic fibers. After curing, polarization, and encapsulation, they obtained 1–3 type lead-free KNLN/PDMS flexible piezoelectric composites, capable of converting mechanical energy into electrical energy for self-powered sensing. Their d_33_g_33_ values reach up to 18 pm^3^/J, comparable to those of traditional lead-containing PZT ceramics, and they store similar amounts of electrical energy under the same mechanical loads. This makes them suitable for replacing lead-containing piezoelectric materials to achieve self-powered sensors.

Traditional power sources have limited energy capacity, making it difficult to sustain a power supply over long periods, and heavy batteries increase the operational burden. When troops are deployed in environments with extreme cold, high altitudes, or low oxygen levels, where conventional power sources are unavailable, they require portable energy harvesting devices to provide a continuous power supply. Shoe-mounted generators offer an alternative energy source for soldiers by utilizing the piezoelectric effect. They convert the mechanical energy from walking into electricity to power electronic devices. Iqra Choudhry et al. [97] developed a shoe-insole nanogenerator (SING) fully integrated into footwear. As a portable energy harvester with no added payload, it scavenges mechanical energy from human walking. The device uses Ecoflex silicone rubber as a flexible matrix, filled with 25 wt% soft PZT nanoparticles and modified with 0.5 wt% graphene nanopowder. Walking tests at a frequency of 2.20 Hz showed an open-circuit peak voltage of 27 V, a short-circuit current of 429.23 µA, a power density of 402 mW/m^2^, and an energy conversion efficiency of approximately 16%. The composite maintained stable output after 10,000 compression cycles. Fabricated from Ecoflex, the insole offers good moisture resistance for long-term stability. However, its output is frequency-dependent, and current applications are limited to energy harvesting. Future optimization aims to achieve a continuous power supply for electronic devices. Chelli et al. [98] prepared flexible piezoelectric composite films by tape casting. The films used polyurethane (PU) as the polymer matrix, lead-free piezoelectric ceramic particles (NKLNT) as fillers, and carbon fibers (CFs) as additives. The purpose was to harvest ambient mechanical vibration energy. This material system is lead-free, so it offers better environmental and safety compatibility for field applications and human wearable scenarios. The PU matrix gives the film flexibility. The film thickness is only 50–100 µm, so it can conform to curved surfaces or body parts without significantly adding to the load. The NKLNT ceramics provide piezoelectric activity. The piezoelectric charge coefficient (d_33_) reaches 147 pC/N. Experiments showed that under periodic mechanical excitation of 20 N at 50 Hz, the composite containing 0.5 wt% carbon fibers achieved an output power of 4.38 µW under load. This output was sufficient to charge a 4.7 µF capacitor and light up a green LED. Although this power level is limited, it is adequate to drive low-power sensors, wireless nodes, or emergency indicator devices. This suggests that such lead-free composite films can serve as an alternative solution for energy harvesting.

Drones have a wide range of applications. In military reconnaissance, drones can avoid exposure risks and adapt more effectively to complex battlefield environments than traditional reconnaissance troops. Among existing energy harvesting technologies for drones, solar energy relies on sunlight, while mechanical energy harvesting is applicable only to specific models. Multirotor drones, due to their low airflow velocity of 8–10 m/s, lack an integrated solution to simultaneously capture wind and vibration energy [99]. Gonçalves et al. [100] Gonçalves et al. [100] integrated a PVDF piezoelectric sensor with an LTC3588-1 power management unit to harvest electrical energy from the propeller-induced wind and motor vibrations of a drone. Experimental results demonstrated that at a motor speed of 3975 rpm, the sensor generated an AC voltage with an amplitude of 17.3 V; this was subsequently rectified and stepped down by the LTC3588-1 chip to deliver a regulated 3.6 V DC output, with an output capacitor charging time of approximately 162 s and a maximum output power of 4 µW. This modular system is capable of independently powering onboard sensors, reducing battery dependence, extending drone flight time, and supporting linear power scaling, making it adaptable to a wide range of application scenarios.

When underwater vehicles are operating, they must accurately determine their distance from other objects to avoid collisions. Pyun et al. [93] reviewed the types of underwater transducers and optimization approaches, as shown in Figure 7b. Underwater transducers can be divided into two major categories: flexure transducers (Classes I–VII) and Tonpilz (mushroom-shaped) transducers. Flexure transducers improve output efficiency by increasing the number of electrodes, essentially stacking multiple PZT elements. Subclasses such as Cymbal (Class V) and Moonie feature curved or slotted end caps, enabling more efficient conversion of the piezoelectric material’s transverse displacement into longitudinal vibration of the end cap. Multi-element arrays enable the piezoelectric activity of each element to work synergistically through proper spacing. Additionally, mounting a mass block at the front of a Tonpilz transducer can lower the device’s resonance frequency.

In recent years, researchers have studied lightweight, highly integrated, slim profile transducers. Their flat, slim profile design reduces acoustic reflections [93,101,102]. Combined with echo control technology, these transducers are suitable for covert integration or lightweight applications, such as underwater vehicle detection and stealth technology, where stealth performance continues to improve. Zhang et al. [101] designed a micromechanical piezoelectric sensor consisting of a PZT film, a substrate with a circular cavity, and interdigitated electrodes. The sensor exhibits a dynamic response without distortion and stable low-frequency performance, with a sensitivity of –172.7 dB (Ref. 1 V/µPa). Its output voltage shows a good linear correlation (r = 0.995) with underwater sound pressure, indicating promising prospects for application in underwater acoustic measurements.

### 5.3. Vibration and Noise Reduction

With the continuous development of industry, noise and vibration issues in sectors such as the automotive and aerospace industries have become increasingly severe. Traditional vibration and noise reduction materials struggle to meet the demands of complex operating conditions. Piezoelectric composite materials, which combine passive energy dissipation with active control capabilities, are widely used in the market and have become core smart materials in active control. However, there is an urgent need to continuously optimize their performance to address practical problems [94,103]. Wang et al. [104] addressed the issue of low-frequency structural noise inside vehicles by applying piezoelectric composites to automotive vibration damping systems. They proposed the DWT-FxLMS algorithm, which integrates the Discrete Wavelet Transform (DWT) with the FxLMS algorithm. They selected the roof panel, which contributes the most to noise, as the active control target and arranged piezoelectric sensors and actuators according to the D-optimization criterion. An experimental platform consisting of an LMS data acquisition system and a dSPACE hardware-in-the-loop simulation system was established for validation. The results showed that this method effectively suppressed roof panel vibrations and reduced the sound pressure level in the 20–200 Hz low-frequency band near the driver’s ear by 2.47 dB(A). This confirms the effectiveness and feasibility of piezoelectric composites in automotive vibration and noise reduction systems.

Aabid et al. [105] reviewed the application of piezoelectric composites in engineering structural control. For vibration control, structural vibration strain energy is converted into electrical energy via the direct piezoelectric effect and absorbed through an RL shunt circuit. For noise control, such as traffic noise energy harvesting, this can be achieved using piezoelectric transducers and supercapacitors. For active control, including damage repair, the inverse piezoelectric effect is used to apply a voltage to PZT patches, thereby generating controllable strain to reduce stress concentrations at crack tips. Zhang et al. [92] prepared a 3D flexible piezoelectric nanofiber aerogel composite consisting of Zn(OAC)_2_/TiBaO_3_/PVDF/PU. This material combines acoustic energy harvesting with full-range noise-reduction capabilities. Within the tested frequency range, the sound absorption coefficient reached a maximum of 0.58, the noise reduction coefficient reached 0.36, and the energy conversion efficiency reached 36%. It also exhibits water repellency (contact angle of 145.4°), thermal insulation (thermal conductivity of 0.0658 W/(m·K)), and good mechanical performance (stress retention exceeding 90% after 100 cycles at 50% strain). These properties offer broad application prospects in energy harvesting and noise reduction in noisy environments such as high-velocity railways, highways, and factories.

## 6. Conclusions

This review summarizes recent advances in improving the piezoelectric properties of piezoelectric composites. It focuses on the intrinsic mechanisms of aligned microstructure and interface design. Structure regulation can be achieved by electric, acoustic, magnetic, and force fields, as well as by template-assisted alignment. These methods align the piezoelectric phase directionally with the matrix and form highly ordered microstructures. Such structures optimize stress transfer, suppress dipole cancellation, and reduce dielectric loss. Interface design includes physical modification, such as high-shear treatment and electrostatic adsorption, and chemical modification, such as silylation, grafting, and amidation. Better interfacial compatibility between the two phases alleviates stress concentration and charge accumulation, improves electromechanical transfer efficiency, and reduces energy loss. The review also introduces the applications of these composites in human motion monitoring, self-powered sensing, vibration and noise reduction, underwater transducers, and portable energy, which indicates promising engineering potential. Future work should target multiscale design from the atomic to the macro level, and the integration of piezoelectricity with optoelectronic, thermoelectric, and sensing functions, so as to broaden applications in flexible electronics, smart sensors, and clean energy.

## Figures and Tables

**Table 1:** Methods and characteristics of aligned microstructure for piezoelectric composites.

Regulation Strategies	Advantages	Disadvantages	Applicability Assessment	Refs.
Electric field	Simple equipment setup; capable of producing fibers with controllable diameters.	Highly sensitive to process parameters; relatively low production efficiency.	Applicable to thin-film and thin-coating devices. Not applicable to thick bulk piezoelectric devices.	[47,48,49,50,51,52]
Acoustic field	Controllable orientation; rapid long-range assembly.	Complicated preparation process; reliance on low-viscosity systems.	No specific requirements on particle chemical composition, shape, or surface properties; incomplete theoretical model.	[53,54]
Magnetic field	Maintains high performance at low filler content; excellent processing controllability.	Requires magnetic particles to enhance magnetic responsiveness; complex process; high cost of strong magnetic field equipment.	Applicable to anisotropic functional composites with tailored properties. Not applicable to high-viscosity systems or large-size components.	[55,56,57]
Force field	Simple process; low cost; suitable for large scale production.	Unidirectional orientation only; difficult to achieve multidirectional orientation; prone to residual stress.	Applicable to tape casting of large-area two-dimensional thin-sheet and thin-film composites. Not applicable to multi-region configurations with different orientations.	[58,59]
Template-assisted alignment	High flexibility; excellent structural controllability.	Complex procedures; high risk of structural damage during post processing.	Applicable to vertically aligned one-dimensional nano-piezoelectric arrays. Limitation: sacrificial templates. Not applicable to large-scale industrial applications.	[60,61]

**Table 2:** Comparison of aligned arrangement methods for piezoelectric composites.

Aligned Arrangement Methods		Material System	Control Sample	d_33_ (pC/N)	g_33_ × 10^−3^ (Vm/N)	Output Voltage (V)	Output Current (µA)	Volume Power Density (mW/cm^3^)	Areal Power Density (µW/cm^2^)	Cyclic Stability	Refs.
Field- assisted alignment	Electric field	0.5 vol%PZT + 0.02 vol%GNP + PDMS	0.5 vol%PZT + 0.02 vol%GNP + PDMS random	17.2 (43.33% ↑)	530 (10.42% ↑)	5.8	4.2	2.3 (64.29% ↑)	-	-	[49]
		37 vol%PZT /PVDF	1.1 vol%PZT /PVDF	22.93 (118.17% ↑)	24.72	0.184	-	-	30.69 (66.43% ↑)	-	[52]
	Acoustic field	γ-glycine/PVA	γ-glycine	10.4 (9.13% ↑)	324						[66]
	Magnetic field	P(VDF-TrFE)/ CoFe_2_O_4_	P(VDF-TrFE)/CoFe_2_O_4_ without magnetic field	33.99 (441% ↑)	-	0.015		0.000438	-	-	[67]
		m-BNNS /PDMS	m BNNS /PDMS random	-		0.806 (100% ↑)	-	-	-	800 times	[56]
	Force field	MXene/ P(VDF-TrFE) DIW	Cast film	-	-	5.5 (80% ↑)	1.1	0.068	-	1000 s	[64]
		BaTiO_3_ NWs/PLA	Cast film			1.4 (86.67% ↑)	0.1645 (84.83% ↑)				[68]
Template-assisted alignment		BaTiO_3_-PDMS Porous	BaTiO_3_-PDMS Dense	-	6.63 (260% ↑)	-	-	-	-		[69]
		BFO-BTO microcone /P(VDF-TrFE)	P(VDF-TrFE)	-	19	5.12 (156% ↑)	-	-	-	Retains 5 V over 3000 cycles.	[70]

The values in parentheses denote the percentage enhancement relative to the control sample. In this table, “↑” denotes an increase (e.g., “10% ↑” means an increase of 10%).

**Table 3:** Methods and characteristics of interface structure design for piezoelectric composites.

Regulation Strategies	Advantages	Disadvantages	Refs.
Silylation	Mature technology; simple process.	Filler agglomeration at high loading; residual impurities.	[71,72,73]
Grafting	High efficiency and simplicity; controllable structure; good stability.	Cumbersome post-treatment; relatively slow reaction rates. Difficult scale-up.	[74,75,76]
Amidation	Good stability; high reaction efficiency; mild conditions.	Specific surface functional group requirements; high byproduct generation; complex post treatment.	[77,78]
Physical Modification	Simple process; environmentally friendly; low costs.	Limited stability; limited durability.	[79,80]

**Table 4:** Comparison of interface structure design methods for piezoelectric composites.

Interface Design Methods		Material System	Control Sample	d_33_ (pC/N)	Output Voltage (V)	Output Current (µA)	Absolute Power (µW)	Cyclic Stability	Refs.
Chemical modification	Silylation	50 wt% PFDTES-BTO /PVDF	50 wt% BTO/PVDF	69.1 (97.43% ↑)	30	1	-	20,000 times	[72]
		TMSPM-PZT /resin	PVDF	25.8	2.3	-	-	-	[73]
	Grafting	10 wt% PMMA@BaTiO_3_ NW/P(VDF-TrFE)	10 wt% BaTiO_3_ NW/P(VDF-TrFE)	-	12.6 (68% ↑)	1.3 (36.84% ↑)	4.25 (120% ↑)	6000 times	[76]
		10 wt% TDPA-PZT /P(VDF-TrFE)	10 wt%PZT /P(VDF-TrFE)	27.1 (22.63% ↑)	3.426 (71.82% ↑)	-	-	After 2000 bending cycles, d_33_ decreased from 27.1 to 26.8 pC/N.	[75]
	Amidation	CNT-COOH:SNA EF40-M/ P(VDF-TrFE)	CNT-COOH /P(VDF-TrFE)	26.4 (8.64% ↑)	2.72 (8.8% ↑)	-	-	-	[78]
		PZT-NH_2_ NPs-SM/SS	Pristine PZT NPs- SM/SS	-	65 (441% ↑)	1.6 (433% ↑)	26	5000 times	[77]
Physical modification		80 wt% BaTiO_3_/PA11 with the solid-state shear milling treatment	Unmilled 80 wt% BaTiO_3_/PA11	4.7 (11.9% ↑)	-	-	-	-	[80]
		5 wt% NaNbO_3_ /P(VDF-TrFE)	5 wt% NaNbO_3_/ P(VDF-TrFE) No laser	-	40 (110.53% ↑)	17.28 (146.86% ↑)	-	After 3000 s cycling, it remained at about 40 V.	[88]

The values in parentheses denote the percentage enhancement relative to unmodified composite films. In this table, “↑” denotes an increase (e.g., “10% ↑” means an increase of 10%).

## Data Availability

No new data were created or analyzed in this study. Data sharing is not applicable to this article.

## References

[1] Habib M., Lantgios I., Hornbostel K. (2022). A review of ceramic, polymer and composite piezoelectric materials. J. Phys. D Appl. Phys..

[2] Wang S., Ma W., Wu J., Chi M., Li T., Wang T., Zhang P. (2021). Preparation technology of 3–3 composite piezoelectric material and its influence on performance. J. Alloys Compd..

[3] Chen L., Chen Y., Ji X. (2024). Fabrication and characterization of barium titanate based 1–3 piezoelectric composites by stereolithography technology. J. Chin. Ceram. Soc..

[4] Raju S.K., Kannan S. (2024). Enhanced building energy harvesting through integrated piezoelectric materials and smart road traffic routing. Lett. Spat. Resour. Sci..

[5] Pan X., Wu Y., Wang Y., Zhou G., Cai H. (2024). Mechanical energy harvesting based on the piezoelectric materials: Recent advances and future perspectives. Chem. Eng. J..

[6] Gao A., Han M., Li B. (2024). Piezoelectric materials used in piezoelectric vibration energy harvesting. J. Phys. Conf. Ser..

[7] Li T., Lee P.S. (2022). Piezoelectric energy harvesting technology: From materials, structures, to applications. Small Struct..

[8] Xiang W., Yin Z., Yang X. (2025). Study and application of PZT in the field of wearable devices. Adv. Mater. Technol..

[9] Leach C., Ali N.K., Hall D.A. (2011). Direct observation of domain switching and crack nucleation in a piezoelectric material. Ceram. Int..

[10] Jiang S., Shen Y., Wang S., Jiang W., Liu Y., Wu Q. (2023). Flexible piezoelectric composite sensor for impact monitoring of curved structures. Sens. Actuators A Phys..

[11] Pandey S.K., James A.R., Raman R., Chatterjee S.N., Goyal A., Prakash C., Goel T.C. (2005). Structural, ferroelectric and optical properties of PZT thin films. Phys. B Condens. Matter.

[12] Holterman J., de Vries T.J.A. (2004). Active damping within an advanced microlithography system using piezoelectric Smart Discs. Mechatronics.

[13] Memarian F., Mohammadi R., Akrami R., Bodaghi M., Fotouhi M. (2023). A comprehensive review of nanocomposite PVDF as a piezoelectric material: Evaluating manufacturing methods, energy efficiency and performance. Nanofabrication.

[14] Chen Z., Chen F., Tu Z., Jiang Q., Wang Y., Liu X., Sheng S. (2025). Microstructure-property relationships in piezoelectric-polymer composites: A review. J. Polym. Res..

[15] Liu X., Xiong C., Sun H., Dong L., Li R., Liu Y. (2006). Piezoelectric and dielectric properties of PZT/PVC and graphite doped with PZT/PVC composites. Mater. Sci. Eng. B.

[16] Cafarelli A., Marino A., Vannozzi L., Puigmartí-Luis J., Pané S., Ciofani G., Ricotti L. (2021). Piezoelectric nanomaterials activated by ultrasound: The pathway from discovery to future clinical adoption. ACS Nano.

[17] Swain S., Kar S.K., Kumar P. (2015). Dielectric, optical, piezoelectric and ferroelectric studies of NBT–BT ceramics near MPB. Ceram. Int..

[18] Rathinasamy S.K., Maheswar R., Lorincz J. (2023). Silk fibroin-based piezoelectric sensor with carbon nanofibers for wearable health monitoring applications. Sensors.

[19] Pu X., Li L., Liu M., Jiang C., Du C., Zhao Z., Hu W., Wang Z. (2016). Wearable self-charging power textile based on flexible yarn supercapacitors and fabric nanogenerators. Adv. Mater..

[20] Aphayvong S., Murakami S., Kanda K., Fujimura N., Yoshimura T. (2022). Enhanced performance on piezoelectric MEMS vibration energy harvester by dynamic magnifier under impulsive force. Appl. Phys. Lett..

[21] Yuan Y., Chen H., Xu H., Jin Y., Chen G., Zheng W., Wang W., Wang Y., Gao L. (2022). Highly sensitive and wearable bionic piezoelectric sensor for human respiratory monitoring. Sens. Actuators A Phys..

[22] Liu M., Zhang L., Qu W., Da L., Liu T. (2025). Advances in MEMS hydrophone technology. IEEE Access.

[23] Selim K.K., Smaili I.H., Yehia H.M., Ahmed M.M.R., Saleeb D.A. (2024). Piezoelectric sensors pressed by human footsteps for energy harvesting. Energies.

[24] Nayak G., Bolla V., Balivada S.K., Prabhudev P. (2022). Technological evolution of ultrasound devices a review. Int. J. Health Technol. Innov..

[25] Lv F., Hong Z., Ahmad Z., Li H., Wu Y., Huang Y. (2023). Design of flexible piezoelectric nanocomposite for energy harvesters: A review. Energy Mater. Adv..

[26] Khan B., Amara U., Khan B., Khan W.U., Ahmad R.U.S., Khan M.S., Hilal M.E., Khoo B.L. (2025). Next-generation piezoelectric materials in wearable and implantable devices for continuous physiological monitoring. Adv. Sci..

[27] Rezvani S., Park S. (2024). Stiffness and damping tuning through using a piezoelectric friction damper and a layered structure. Smart Mater. Struct..

[28] (1988). IEEE Standard on Piezoelectricity.

[29] Su Y., Li W., Cheng X., Zhou Y., Yang S., Zhang X., Chen C., Yang T. (2022). High-performance piezoelectric composites via β phase programming. Nat. Commun..

[30] Wu C., Gao X., Zhu H., Wang X., Wang S., Fu Y., Han Y., Yuan W., Tian Y., Jiang X. (2025). Multiscale stress transmission regulation toward flexible strain sensors with high stability. Adv. Funct. Mater..

[31] Zeng H., Xu Q., Zhang J., Zhou P., Zhang J., Li J., Zhao S., Zhou K., Zhang D., Bowen C. (2026). Topology-optimized stretchable piezoelectric sensors with tailored liquid-metal circuits for anisotropic stress-adaptive motion monitoring. Adv. Mater..

[32] Yuan X., Yan A., Lai Z., Liu Z., Yu Z., Li Z., Cao Y., Dong S. (2022). A poling-free PVDF nanocomposite via mechanically directional stress field for self-powered pressure sensor application. Nano Energy.

[33] Shao Z., Zhang X., Liu J., Liu X., Zhang C. (2023). Electrospinning of highly bi-oriented flexible piezoelectric nanofibers for anisotropic-responsive intelligent sensing. Small Methods.

[34] Guo Y., Zhang H., Zhong Y., Shi S., Wang Z., Wang P., Zhao Y. (2023). Triboelectric nanogenerator-based near-field electrospinning system for optimizing pvdf fibers with high piezoelectric performance. ACS Appl. Mater. Interfaces.

[35] Du X., Zhou Z., Zhang Z., Yao L., Zhang Q., Yang H. (2022). Porous, multi-layered piezoelectric composites based on highly oriented PZT/PVDF electrospinning fibers for high-performance piezoelectric nanogenerators. J. Adv. Ceram..

[36] Qi W., Li L., Han R., Hou Y., Zhou Z., Chen G.-X., Li Q. (2024). Enhancing dielectric properties of (CaCu_3_Ti_4_O_12_ NWs-Graphene)/PVDF ternary oriented composites by hot stretching. ACS Omega.

[37] Tang T., Shen Z., Wang J., Xu S., Jiang J., Chang J., Guo M., Fan Y., Xiao Y., Dong Z. (2023). Stretchable polymer composites with ultrahigh piezoelectric performance. Natl. Sci. Rev..

[38] Kwak M.S., Park Y.J., Kim M.P., Ko H. (2025). Interfacial polarization for high-performance triboelectric devices: Principles, strategies, and applications. ACS Appl. Mater. Interfaces.

[39] Lu H., Liu X., Burton J.D., Bark C.W., Wang Y., Zhang Y., Kim D.J., Stamm A., Lukashev P., Felker D.A. (2012). Enhancement of ferroelectric polarization stability by interface engineering. Adv. Mater..

[40] Liu L., Zhang H., Zhou S., Du C., Liu M., Zhang Y. (2024). Boosting the piezoelectric response and interfacial compatibility in flexible piezoelectric composites via DET-Doping BT nanoparticles. Polymers.

[41] Wang L., Yang J., Cheng W., Zou J., Zhao D. (2021). Progress on polymer composites with low dielectric constant and low dielectric loss for high-frequency signal transmission. Front. Mater..

[42] Zhang Y., Chen J., Yu R., Liu S., Qin Y. (2025). High-performance piezotronic devices. ACS Nano.

[43] Shao M., Wang C., Zheng T., Liang J. (2026). An acoustofluidic capillary nozzle for programmable microstructure assembly in direct ink writing of flexible conductive composites. Micromachines.

[44] Wang B., Du M., Wang H., Wang M., Li D. (2025). Giant modulation of microstructure and ferroelectric/piezoelectric responses in Pb(Zr,Ti)O_3_ ultrathin films via single-pulse femtosecond laser. Nanomaterials.

[45] Jang H.S., Heo I.B., Baek C., Jeon D.W., Lee D., Alluri N.R., Choi H., Jeon H.M., Kim S.H., Kim H. (2026). In situ amidation-derived interfacial modulation for homogeneous ultra-high nanoparticle loading toward robust and flexible piezoelectric composites. Adv. Funct. Mater..

[46] Nguyen T.T., Phan T.T.M., Chu N.C., Boï Luu V., Nguyen X.H., Martin I., Carriere P. (2014). Elaboration and dielectric property of modified PZT/epoxy nanocomposites. Polym. Compos..

[47] Shao H., Fang J., Wang H., Lin T. (2015). Effect of electrospinning parameters and polymer concentrations on mechanical-to-electrical energy conversion of randomly-oriented electrospun poly(vinylidene fluoride) nanofiber mats. RSC Adv..

[48] Zaarour B., Zhu L., Jin X. (2019). Controlling the surface structure, mechanical properties, crystallinity, and piezoelectric properties of electrospun PVDF nanofibers by maneuvering molecular weight. Soft Mater..

[49] Yildirim A., Grant J.C., Shams Es-haghi S., Lee W., Maruthamuthu M.K., Verma M., Sutherland J.W., Cakmak M. (2020). Roll-to-roll (R2R) production of large-area high-performance piezoelectric films based on vertically aligned nanocolumn forests. Adv. Mater. Technol..

[50] Wilson S.A., Maistros G.M., Whatmore R.W. (2005). Structure modification of 0-3 piezoelectric ceramic/polymer composites through dielectrophoresis. J. Phys. D Appl. Phys..

[51] D’Ambrogio G., Zahhaf O., Hebrard Y., Le M.Q., Cottinet P.J., Capsal J.F. (2020). Micro-structuration of piezoelectric composites using dielectrophoresis: Toward application in condition monitoring of bearings. Adv. Eng. Mater..

[52] Chamankar N., Khajavi R., Yousefi A.A., Rashidi A., Golestanifard F. (2020). A flexible piezoelectric pressure sensor based on PVDF nanocomposite fibers doped with PZT particles for energy harvesting applications. Ceram. Int..

[53] Johnson K., Melchert D., Gianola D.S., Begley M., Ray T.R. (2021). Recent progress in acoustic field-assisted 3D-printing of functional composite materials. MRS Adv..

[54] Lu L., Tang X., Hu S., Pan Y. (2018). Acoustic field-assisted particle patterning for smart polymer composite fabrication in stereolithography. 3D Print. Addit. Manuf..

[55] Zeng Y., Bao G., Yi J., Zhang G., Jiang S. (2015). The influence of inducing magnetic field on the magnetoelectric effect of particulate magnetoelectric composites. J. Alloys Compd..

[56] Xin C., Zhao H., Chen X., Shi C., Ma D., Song Q., Zhao Q., Zhang J., Wang C. (2024). Flexible piezoelectric polymer composites with magnetic-field-oriented BNNS and imprinted micropillars for self-powered sensors. Natl. Sci. Open.

[57] Kim T. (2022). Characteristics of a 2-2 piezoelectric composite transducer made by magnetic force assembly. Crystals.

[58] Chen X., Shao J., An N., Li X., Tian H., Xu C., Ding Y. (2015). Self-powered flexible pressure sensors with vertically well-aligned piezoelectric nanowire arrays for monitoring vital signs. J. Mater. Chem. C.

[59] Hou L., Huan Y., Zheng M., Liu Y., Wang C., Wang X., Li C., Wang Z., Wei T. (2024). 3D vertically aligned microchannel structure to enhance piezoelectric energy harvesting performance of PZT/PVDF&CNTs piezoelectric composites. J. Mater..

[60] Zhang H., Zhou M., Zhao H., Lei Y. (2021). Ordered nanostructures arrays fabricated by anodic aluminum oxide (AAO) template-directed methods for energy conversion. Nanotechnology.

[61] Jung W.S., Do Y.H., Kang M.G., Kang C.Y. (2013). Energy harvester using PZT nanotubes fabricated by template-assisted method. Curr. Appl. Phys..

[62] Lu M., Zeng L., Jiang C., Li Q., Guo Y. (2025). Highly sensitive and omnidirectional anisotropic piezoelectric sensors based on oriented PbZr_0.52_Ti_0.48_O_3_ fibers with intrinsic flexibility. J. Alloys Compd..

[63] Mohammadi B., Yousefi A.A., Bellah S.M. (2007). Effect of tensile strain rate and elongation on crystalline structure and piezoelectric properties of PVDF thin films. Polym. Test..

[64] Li C., Huang Z., Zhang L., Song Z., Chen Y. (2024). 3D printing enhanced piezoelectricity of MXene/P(VDF-TrFE) composites for energy harvesting and force sensing. Compos. Sci. Technol..

[65] Zhang Y., Xie M., Roscow J., Bao Y., Zhou K., Zhang D., Bowen C.R. (2017). Enhanced pyroelectric and piezoelectric properties of PZT with aligned porosity for energy harvesting applications. J. Mater. Chem. A.

[66] Xue H., Jin J., Tan Z., Chen K., Lu G. (2024). Flexible, biodegradable ultrasonic wireless electrotherapy device based on highly self-aligned piezoelectric biofilms. Sci. Adv..

[67] Fortunato M., Bidsorkhi H.C., D’Aloia A.G., Tamburrano A., Sarto M.S. Potential Application of PVDF/CoFe_2_O_4_ Nanocomposites as Self-powered Piezoelectric Plantar Pressure Sensors. Proceedings of the 2023 IEEE International Symposium on Medical Measurements and Applications (MeMeA).

[68] Malakooti M.H., Julé F., Sodano H.A. (2018). Printed Nanocomposite Energy Harvesters with Controlled Alignment of Barium Titanate Nanowires. ACS Appl. Mater. Interfaces.

[69] Su X., Liao W., Fang F. (2025). Combining oriented ceramic skeleton and porous PDMS towards high performance flexible piezoelectric energy harvester. Compos. Sci. Technol..

[70] Yang W., Zhan H., Lv P., Gao G., Zhang S., Xin L., Li C., Ren L., Qian J., Zhang M. (2026). Flexible pyramidal BiFeO_3_–BaTiO_3_ microcone arrays integrated with P(VDF-TrFE) for self-powered mechanical sensing and energy harvesting. Nano Res..

[71] Wu J., Ma W., Chi M., Wang S., Zhang P. (2020). Effect of surface modification of ferroelectric ceramic component on the properties of PZT-type/epoxy piezoelectric composite with spiral structure. J. Alloys Compd..

[72] Li H., Song H., Long M., Saeed G., Lim S. (2021). Mortise-tenon joint structured hydrophobic surface-functionalized barium titanate/polyvinylidene fluoride nanocomposites for printed self-powered wearable sensors. Nanoscale.

[73] Jin L., Song J., Liu L., Jia Y. (2024). PZT-based flexible piezoelectric sensors for real-time condition monitoring. AIP Adv..

[74] Gebrekrstos A., Kar G.P., Madras G., Misra A., Bose S. (2019). Does the nature of chemically grafted polymer onto PVDF decide the extent of electroactive β-polymorph?. Polymer.

[75] Chen J., Li J., Cheng C., Chiu C. (2022). Piezoelectric Property Enhancement of PZT/Poly(vinylidenefluoride-co-trifluoroethylene) Hybrid Films for Flexible Piezoelectric Energy Harvesters. ACS Omega.

[76] Shi K., Chai B., Zou H., Shen P., Sun B., Jiang P., Shi Z., Huang X. (2021). Interface induced performance enhancement in flexible BaTiO_3_/PVDF-TrFE based piezoelectric nanogenerators. Nano Energy.

[77] Lee E.J., Kim T.Y., Kim S.-W., Jeong S., Choi Y., Lee S.Y. (2018). High-performance piezoelectric nanogenerators based on chemically-reinforced composites. Energy Environ. Sci..

[78] Li J., Huang C., Chen K., Chen J., Hsu X., Chen Y. (2020). Enhanced piezoelectric properties of Poly(Vinylidenefluoride-Co-Trifluoroethylene)/carbon-based nanomaterial composite films for pressure sensing applications. Polymers.

[79] Mahato P.K., Sen S. (2015). Effect of surface modification of ceramic particles by SDS on the electrical properties of PZT-PVDF and BT-PVDF composites: Interface effect. J. Mater. Sci. Mater. Electron..

[80] Qi F., Chen N., Wang Q. (2017). Preparation of PA11/BaTiO_3_ nanocomposite powders with improved processability, dielectric and piezoelectric properties for use in selective laser sintering. Mater. Des..

[81] Kumar S., Thakur A., Mishra A.K., Kumar R., Selvaraj M., Karnwal A., Kumari D., Srivastava A., Khan U.A., Asthana N. (2025). Materials and processes in nanogenerators for energy harvesting: A review of chemical modification strategies and paradigms. J. Alloys Compd..

[82] Zhang W., Zhang Y., Yan X., Hong Y., Yang Z. (2023). Challenges and progress of chemical modification in piezoelectric composites and their applications. Soft Sci..

[83] Yuan Z., Li C., Gong Y., Zhou H., Liu Y. (2026). Large piezoelectricity in crosslinked ferroelectric polymers. Nat. Commun..

[84] Bouad V., Girardot M., Ladmiral V., Barrau S. (2024). Piezoelectric fluorinated polymer composites: A review on coupling agents at the filler/matrix interface. Polym. Compos..

[85] Yuan Z., Tong H., Fei Z., Li C., Liu Y. (2025). Scalable solution-processed ferroelectric polymers exhibiting markedly enhanced piezoelectricity. Nat. Commun..

[86] Cao J., Zhou W., Li T., Guan Y., Liu J., Zhao Y., Li Y. (2023). Interfacial engineering of PZT/PVDF composites via insulating MgO as an interlayer towards enhanced dielectric performances. J. Polym. Res..

[87] Zhou Z., Zhang Q., Zhang Z., Kuang H., Du X., Yang H. (2022). Lead-free, high-current output piezoelectric nanogenerators using three-dimensional interdigitated electrodes. Chem. Eng. J..

[88] Liu Y., Wang F., Chen X., Cui F., Lu Y., Gao R., Yu L. (2026). Enhanced output performance of NaNbO_3_/P(VDF-TrFE)-based piezoelectric nanogenerators by femtosecond laser surface modification for bioenergy harvesting and human motion monitoring. ACS Sustain. Chem. Eng..

[89] Cao X., Xiong Y., Sun J., Zhu X., Sun Q., Wang Z. (2021). Piezoelectric nanogenerators derived self-powered sensors for multifunctional applications and artificial intelligence. Adv. Funct. Mater..

[90] Yang Y., Pan H., Xie G., Jiang Y., Chen C., Su Y., Wang Y., Tai H. (2019). Flexible piezoelectric pressure sensor based on polydopamine-modified BaTiO_3_/PVDF composite film for human motion monitoring. Sens. Actuators A Phys..

[91] Jadidian B., Marandian Hagh N., Winder A.A., Safari A. (2009). 25 MHz ultrasonic transducers with lead- free piezoceramic, 1-3 PZT fiber-epoxy composite, and PVDF polymer active elements. IEEE Trans. Ultrason. Ferroelectr. Freq. Control.

[92] Zhang Z., Li Z., Li Q., Liu X., Gong J., Li H. (2025). A PVDF/PU-based composite 3D flexible piezoelectric nanofiber aerogel for acoustic energy harvesting and noise reduction. Chem. Eng. J..

[93] Pyun J.Y., Kim Y.H., Park K.K. (2023). Design of piezoelectric acoustic transducers for underwater applications. Sensors.

[94] Zhou J., Li Z., Zhou J., Song R., Wu S., Zhu Y., He J. (2026). Interfacial Modification of Macro Fiber Composites for Active Low-Frequency Vibration Suppression. Materials.

[95] Singh D., Choudhary A., Garg A. (2017). Flexible and robust piezoelectric polymer nanocomposites based energy harvesters. ACS Appl. Mater. Interfaces.

[96] Stuber V.L., Deutz D.B., Bennett J., Cannel D., de Leeuw D.M., van der Zwaag S., Groen P. (2018). Flexible lead-free piezoelectric composite materials for energy harvesting applications. Energy Technol..

[97] Choudhry I., Khalid H.R., Lee H.-K. (2020). Flexible piezoelectric transducers for energy harvesting and sensing from human kinematics. ACS Appl. Electron. Mater..

[98] Chelli Z., Achour H., Saidi M., Laghrouche M., Chaouchi A., Rguiti M., Lorgouilloux Y., Courtois C. (2021). Fabrication and characterization of PU/NKLNT/CFs based lead-free piezoelectric composite for energy harvesting application. Polym.-Plast. Technol. Mater..

[99] Liu Q., Li Q., Fang Z., Zhou X., Wang R., Zuo C. (2021). Piezoelectric energy harvesting for flapping wing micro air vehicle and flapping wing sensing based on flexible polyvinylidene fluoride. Appl. Sci..

[100] Gonçalves L.S., Pereira R.M.L., Tyszler R.S., Morais M.C.A.M., Barbosa C.R.H. (2025). Application of PVDF transducers for piezoelectric energy harvesting in unmanned aerial vehicles. Energies.

[101] Zhang X., Cui Z., Wu H., Luo J., Ye T., Shan X., Xie T., Ma B. (2024). Micromachined piezoelectric sensor with radial polarization for enhancing underwater acoustic measurement. Compos. Struct..

[102] Wu X., Peng Y., Li S., Zheng H., Zhang Z. (2025). A miniaturized high-sensitivity hydroacoustic transducers based on P(VDF-TrFE) piezoelectric materials. IEEE Sens. J..

[103] Yang Z., Zhang J., Gao D., Liu K., Wu Z. (2017). Advance of aerospace smart material and structure. Aeronaut. Manuf. Technol..

[104] Wang Y.S., Guo H., Li Y.R., Liu N.N., Yang C. (2020). Active control for vehicle interior noise based on DWT-FxLMS algorithm using a piezoelectric feedback system. Appl. Acoust..

[105] Aabid A., Parveez B., Raheman M.A., Ibrahim Y.E., Anjum A., Hrairi M., Parveen N., Mohammed Zayan J. (2021). A review of piezoelectric material-based structural control and health monitoring techniques for engineering structures: Challenges and opportunities. Actuators.

